# Systematic review of preterm birth multi-omic biomarker studies

**DOI:** 10.1017/erm.2022.13

**Published:** 2022-04-05

**Authors:** Juhi K. Gupta, Ana Alfirevic

**Affiliations:** 1 Wolfson Centre for Personalised Medicine, Department of Pharmacology and Therapeutics, Institute of Systems, Molecular and Integrative Biology, University of Liverpool, Liverpool L69 3GL, UK; 2 Harris-Wellbeing Research Centre, University Department, Liverpool Women's Hospital, Liverpool L8 7SS, UK

**Keywords:** Epigenetics, genomics, maternal biomarkers, metabolomics, multi-omics, omics, preterm birth, preterm labour, proteomics, transcriptomics

## Abstract

Preterm birth (PTB) is one of the leading causes of deaths in infants under the age of five. Known risk factors of PTB include genetic factors, lifestyle choices or infection. Identification of omic biomarkers associated with PTB could aid clinical management of women at high risk of early labour and thereby reduce neonatal morbidity. This systematic literature review aimed to identify and summarise maternal omic and multi-omic (genomics, transcriptomics, proteomics and metabolites) biomarker studies of PTB. Original research articles were retrieved from three databases: PubMed, Web of Science and Science Direct, using specified search terms for each omic discipline. PTB studies investigating genomics, transcriptomics, proteomics or metabolomics biomarkers prior to onset of labour were included. Data were collected and reviewed independently. Pathway analyses were completed on the biomarkers from non-targeted omic studies using Reactome pathway analysis tool. A total of 149 omic studies were identified; most of the literature investigated proteomic biomarkers. Pathway analysis identified several cellular processes associated with the omic biomarkers reported in the literature. Study heterogeneity was observed across the research articles, including the use of different gestation cut-offs to define PTB. Infection/inflammatory biomarkers were identified across majority of papers using a range of targeted and non-targeted approaches.

## Introduction

‘Omics’ refers to the biosciences fields with the suffix ‘-omics’, such as genomics, transcriptomics, metabolomics and proteomics, which are commonly explored for biomarkers of diseases. Preterm birth (PTB), defined by the World Health Organisation (WHO) as birth less than 37 weeks of gestation, is a complex multi-factorial condition with no robust biomarkers. A higher rate of morbidity in neonates is associated with ‘very preterm’ births (between 28 and 32 weeks of gestation) compared with ‘moderate to late preterm’ births (32 to 37 weeks of gestation) (Refs 1, 2). Non-medically indicated PTB can be further categorised into the clinical sub-groups: spontaneous preterm labour with intact membranes (sPTB) and preterm pre-labour rupture of membranes (PPROM).

Risk factors of PTB include genetics, lifestyle choices and environmental influences (infection, nutrition or maternal stress) (Ref. 3). Studies have shown that women with a family history of PTB in mothers or sisters are at higher risk of delivering preterm (Refs 4–7). Svensson *et al*. (Ref. 7) reported that 25% of variance in PTB was explained by maternal genetic effects. PTB risk has also been shown to vary between different ethnic groups (Ref. 8).

A more personalised approach of screening women at risk of PTB is required to improve pregnancy outcomes, and hence reduce rates of PTB, as opposed to the traditional methods that are currently used (such as collecting obstetric history or ultrasound scans) (Ref. 3). In the aim to identify molecular biomarkers involved in the onset of PTB and utilise these for diagnosis, many types of omic studies have been conducted (Ref. 9).

Several genes involved in the inflammatory pathway, such as *EBF1* (early B-cell factor 1), and various signalling molecules, for instance Wnt, have been indicated as potential biomarkers of PTB in the literature (Ref. 10). However, the mechanisms of action are not yet understood. Recent studies have demonstrated that the genomes of large populations can be screened using advanced microarray technology, enhancing biomarker research (Refs 10–12).

In addition to genomic studies, gene expression level analyses (or the field of ‘transcriptomics’) using microarray or sequencing technology have highlighted more candidate biomarkers including *ABCA13* (ATP-binding cassette sub-family A member 13) reported by Heng *et al*. (Ref. 13). More recently, microRNAs (miRNA) and miR (mature form of miRNA) have been associated with PTB (Refs 14–18). miRNAs are non-coding RNAs with a key role in gene expression regulation (Ref. 19). Gene expression can be measured between different gestational time points and sample types, enabling longitudinal analysis. Many protein biomarkers of PTB such as MMP-8 (matrix metalloproteinase-8), TNF (tumour necrosis factor) and interleukins (ILs) have been identified using a range of omic technologies (Refs 20–22).

Metabolites are small molecules that are part of primary and intermediate metabolism that can help unveil the underlying pathophysiology of early labour (Refs 23, 24). Mass spectrometry and nuclear magnetic resonance (NMR), traditionally applied in proteomics studies, have been implemented for in-depth metabolome exploration for PTB biomarker discovery (Refs 25, 26).

Different approaches have been implemented for omic studies such as targeted and non-targeted (or unbiased) techniques. Unbiased methods have enhanced exploration of the genome, transcriptome, proteome and metabolome enabling detection of candidate biomarkers related to a disease (Refs 10, 15, 24).

The pathways involved with the onset of sPTB are not yet known, even though several omic studies have suggested different markers of PTB (or sPTB or PPROM). Multi-omic data analysis approaches could assist with (1) identification of the mechanistic pathways initiated in PTB, by exploring interactions between different types of omic data; (2) improve our understanding of the ‘systems biology’ of PTB and (3) determine biomarkers in early stages of pregnancy for earlier and more effective clinical intervention to improve pregnancy outcome and neonatal health outcome. A summary of candidate omic biomarkers could aid future PTB omic studies, particularly multi-omic research. This systematic literature review aimed to (1) identify and summarise maternal omic (genomics, transcriptomics, proteomics and metabolites) biomarker studies of PTB, including the phenotypes sPTB and PPROM and (2) of these studies, identify which studies performed multi-omic research.

## Methods

Literature searches were conducted using three databases: PubMed, Web of Science and Science Direct. The resulting literature studies were filtered for journal (research) articles and human studies only. Original research articles from individual omic fields, published up to and including November 2020, were identified using the following search terms:

1. *Genomics*: ((Preterm birth OR preterm labour OR preterm delivery OR pregnancy OR premature birth OR premature labour OR premature delivery)) AND (biomarkers OR bioindicators OR biological markers OR biochemical markers OR predictors) AND (genomics OR genetics OR genome wide association OR GWAS OR genetic associations OR DNA OR single nucleotide polymorphisms OR epigenetic OR epigenome OR methylation).
2. *Transcriptomics*: ((Preterm birth OR preterm labour OR preterm delivery OR pregnancy OR premature birth OR premature labour OR premature delivery)) AND (biomarkers OR bioindicators OR biological markers OR biochemical markers OR predictors) AND (transcriptomics OR RNA OR mRNA OR miRNA OR microRNA OR gene expression profiling OR RNA profiling OR microRNA profiling OR transcriptome).
3. *Proteomics*: ((Preterm birth OR preterm labour OR preterm delivery OR pregnancy OR premature birth OR premature labour OR premature delivery)) AND (biomarkers OR bioindicators OR biological markers OR biochemical markers OR predictors) AND (proteomic OR clinical proteomics OR proteins OR protein profiling OR peptidomic profiling OR proteome).
4. *Metabolomics*: ((Preterm birth OR preterm labour OR preterm delivery OR pregnancy OR premature birth OR premature labour OR premature delivery)) AND (biomarkers OR bioindicators OR biological markers OR biochemical markers OR predictors) AND (metabolomics OR metabolites OR metabolome).

Literature was screened and selected if the following inclusion criteria were met: an original research article investigating PTB (including PPROM and sPTB of any gestational age); maternal sample (human only) was analysed (including whole blood, plasma, serum, cervical vaginal fluid and amniotic fluid); the sample was taken before start of labour process or symptoms and was a biomarker study with omic methods or analysis were indicated. Research of both foetal and maternal samples was included but only maternal analysis results were reported in this review (if maternal analysis could be separated from foetal analysis).

Studies were excluded if samples were collected after patients were admitted to hospital because of presenting symptoms of PTB (such as start of contractions or ruptured membranes observed) or if the births were iatrogenic (medically induced). Genetic studies investigating familial relative inheritability of genes associated with PTB were excluded as no direct comparisons between term and PTB outcomes based on maternal genes.

Papers that reported investigation of more than one omic dataset were allocated to one omic systematic search to avoid duplication.

Research articles that met the review criteria were summarised into targeted studies (where a select number of candidate markers was chosen for exploration) or non-targeted investigations (whereby the whole genome, transcriptome, proteome or metabolome was explored) based on reported methods and technologies applied.

Biomarkers reported in the literature from non-targeted approaches were collated per omic field (gene symbols, miRNA names, UniProt IDs and KEGG IDs). Duplicates were removed and each omic list was uploaded to Reactome for pathway analyses (Refs 27, 28). Literature investigating more than one type of omic data was identified. PTB gestation cut-offs applied in all identified literature studies were plotted using the ‘Matplotlib’ v3.3.3 package in Python 3.8.

## Results

**Fig. 1.**
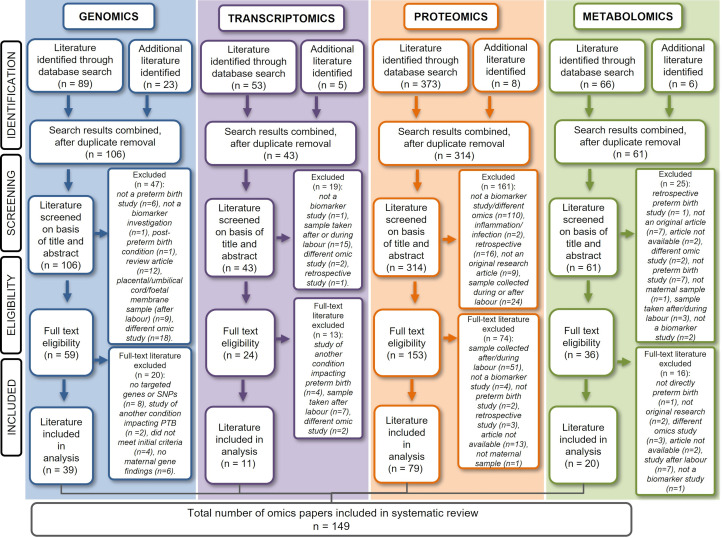
PRISMA diagrams of individual PTB omic biomarker literature searches (*N* = 149) (last search date: 18/06/2021).

PRISMA diagrams of the individual omic systematic searches are summarised in Figure 1. In total, 149 omic studies were identified, of which the majority were proteomics biomarker studies (*n* = 79), followed by genomics (*n* = 39), metabolomics (*n* = 20) and transcriptomics research (*n* = 11).

### Genomic biomarkers

Of the 39 genomic studies identified, nine applied non-targeted approaches (including microarrays) (Table 1), whereas 30 utilised targeted (mainly polymerase chain reaction methods) (Table S1).

**Table 1.** Maternal genes or SNPs identified in PTB (or PTL for preterm labour) unbiased genome-wide screen studies (*n* = 9)

Study	Ethnic groups	Genes/SNPs identified	Phenotype	Total number of maternal participants	Technology/technique	*P* value
Rappoport *et al*. (Ref. 12)	European, African, American, Asian	rs1979081 (upstream of FAM87A) – Americas rs17591250 – African	PTB (<37 weeks)	888 [PTB *n* = 334, control *n* = 554]	Illumina HumanOmni 2.5 array	*P* < 0.05
Zhang *et al*. (Ref. 10)	European	**Gestational age:** *EBF1 (*rs2963463, rs2946171) *EEFSEC* (rs2955117, rs200745338) *AGTR2* (rs201226733, rs5950491) *WNT4* (rs56318008, rs12037376) *ADCY5* (rs4383453, rs9861425) *RAP2C* (rs200879388) **PTB:** *EBF1* (rs2963463, rs2946169) *EEFSEC* (rs201450565, rs200745338) *AGTR2* (rs201386833, rs5950506)	Gestational age and PTB (<37 weeks)	43 568	Illumina HumanHap550+ BeadChip	Findings reported: *P* < 1 × 10^−8^
Manuck *et al*. (Ref. 29)	African American women	SNPs within 7q21-7q31 region (PTB susceptibility) and LOD >1.5: rs7802588, rs7807771, rs10488004, rs6465387, rs10256829, rs6466115, rs2790012, rs1322849, rs9784907, rs883403, rs17154865, rs1525200, rs10275038, rs12533049, rs2141360	sPTB (<37 weeks)	177	Illumina African American admixture panel	NA
Bacelis *et al*. (Ref. 30)^a^	Norwegian - European	*NFKB1* (rs1609798) *ENG* (rs10117075) *MEFV* (rs220381) *SP3* (rs6718188) *ABCA1* (rs12336969) *DPY19L2* (rs1607800) *FRMD4A* (rs3740121)	Gestational age and PROM (PTB, <37 weeks)	1921	Association tests and gene-set enrichment analysis	NA
Zhang *et al*. (Ref. 31)^a^	Caucasian, African American, Hispanic, Other	*DCP1A* (rs17053026) *GLIS3* (rs501631) *SPNS3* (rs7211542) *CNTN5* (rs1025888)	sPTB (<34 weeks)	2210 [PTB *n* = 935, control *n* = 946, validation cohort control *n* = 329]	Affymetrix genome-wide human SNP array	NA
Wu *et al*. (Ref. 32)	European	544 675 SNPs on autosomes analysed	sPTB (<37 weeks)	2000 [PTB *n* = 1000, control *n* = 1000]	Illumina Human660W quad array	NA
Myking *et al*. (Ref. 33)	American, Danish and Norwegian	rs4562494 rs7892483 rs5972070 *FRMD7* (rs2747022) *IL1RAPL1* (rs4829104) *REPS2* (rs12557633)	sPTB (<37 weeks)	3022 [PTB *n* = 1535, control *n* = 1487]	Illumina Human660W quad array	NA
Karjalainen *et al*. (Ref. 34)	Finnish	*AR* and *IL2RG*	sPTB (<36 weeks)	473 [PTB *n* = 272, control *n* = 201]	Affymetrix genome-wide human SNP array	NA
Plunkett *et al*. (Ref. 35)	European American, African American and Hispanic (Mexican)	*FSH* rs6741370 *FSHR* (rs11686474, rs11680730, rs12473815)	PTB (37 weeks)	328 [PTB *n* = 165, control *n* = 163]	Affymetrix genome-wide human SNP array	NA

LOD score, logarithm of the odds score; PTB, preterm birth; SNP, single nucleotide polymorphisms; sPTB, spontaneous preterm birth.

Where available, relevant significant *P*-value cut-off have been summarised.

a Summarised top SNPs (*P* < 1 × 10^−4^) associated with genes.

Many single-nucleotide polymorphisms (SNPs) across different ethnicities were identified as associated with PTB or sPTB (Table 1). In European ethnic populations, *EBF1* and *NFKB1* (nuclear factor kappa B subunit 1), both of which are inflammation-related (Refs 10, 30) (Table 1). Zhang *et al*.'s (Ref. 10) genome-wide association study identified *EBF1* as significantly associated with PTB and gestational age. EBF1 transcripts were further explored by Zhou *et al*. (Ref. 18).

Inflammation- and infection-associated genes *TIMP2* (tissue inhibitor of metalloproteinase 2) (rs2277698), *COL4A3* (collagen type IV alpha 3 chain) (rs1882435) and *TNF* variants were explored in more than one targeted study (Table S1). Frey *et al*. (Ref. 36) and Romero *et al*. (Refs 5, 37) examined *TIMP2*. Similarly, *COL4A3* was reported by Romero *et al*. (Refs 5, 37). *TNF*, a pro-inflammatory cytokine, was investigated by Annells *et al*. (Ref. 38), Gebhardt *et al*. (Ref. 39), Ramos *et al*. (Ref. 40) and Valdez *et al*. (Ref. 41) (Table S1). Similarly, *TNF*-*α* was reported by Fortunato *et al*. (Ref. 42) and Menon *et al*. (Refs 22, 43).

Fortunato *et al*. (Ref. 42), Menon *et al*. (Ref. 22) and Plunkett and Muglia (Ref. 7) suggested an association between PTB and TNF receptors 1 (TNFR1) and 2 (TNFR2) genes. Kalish *et al*. (Ref. 44) found that *TNFRSF6* (tumour necrosis factor receptor superfamily member 6 or Fas gene) was associated with an increased risk of PPROM. Toll-like receptors are proinflammatory agonists, gene variants of which were associated with an increased risk of PPROM as reported by Ramos *et al*. (Ref. 40) and Romero *et al*. (Ref. 36) (Table S1).

No epigenetic studies that met the criteria were identified, however Zhang *et al*. (Ref. 10) detected H3K4me3 (histone H3 lysine 4 trimethylation) in the endometrial stromal cell line. Zhang *et al*. (Ref. 10) suggested that the binding of EBF1 to the SNP region rs3820282 modulates *WNT4*, which could influence gestational length.

### Transcriptomics biomarkers

Fewer transcriptomics studies met the review criteria, five were non-targeted (Table 2) and six were targeted biomarker studies (Table S2).

**Table 2.** Summary of PTB non-targeted transcriptomics biomarker literature (*n* = 5) that investigated maternal samples

Study	Transcripts identified	Phenotype	Sample type	Total number of maternal participants	Technology/technique	*P* value
Cook *et al*. (Ref. 45)	hsa-let-7a-5p, hsa-miR-374a-5p, hsa-miR-15b-5p, hsa-miR-19b-3p, hsa-miR-23a-3p, hsa-miR-93-5p, hsa-miR-150-5p, hsa-miR-185-5p and hsa-miR-191-5p	sPTL <34 weeks	Plasma	164 [PTB *n* = 23]	nCounter miRNA assay	*P* < 0.001
Gray *et al*. (Ref. 16)	miR223 (increase in sPTB) miR302b, miR1253, clustering of miR548 miRNAs [miR548a, miR548aa, miR548ai, miR548ak and miR548n] (under expressed in sPTB)	sPTL (28–32 weeks)	Plasma	24 [sPTL *n* = 12, control *n* = 12]	Nanostring nCounter miRNA expression assay	*P* < 0.001
Heng *et al*. (Ref. 13)	**SPTB genes up-regulated:** *ABCA13*, *MYOF*, *SASH1*, *LAP3* **SPTB genes down-regulated:** *FCER1A*, *CPA3*, *ABCG1*, *ABCA1*	sPTB (<37 weeks)	Blood	165 [PTB *n* = 51; control *n* = 114]	Affymetrix human gene 2.1 ST array and RT-PCR	*P* < 0.001
Sanders *et al*. (Ref. 17)	miR-21, miR-142, miR-30e, miR-148b, miR-29b and miR-223	PTB (<37 weeks)	Cervical cells	53 [PTB *n* = 4, control *n* = 49]	NanoString nCounter miRNA expression assay	*P* = 0.009
Elovitz *et al*. (Ref. 15)	miR-25, miR-145, miR-143, miR-199b-5P	PTB (<37 weeks)	Cervical cells	20 [PTB *n* = 10, control *n* = 10]	Affymetrix GeneChip miRNA array	*P* < 0.002

miR, microRNA; PTB, preterm birth; RT-PCR, reverse transcriptase-polymerase chain reaction; sPTB, spontaneous preterm birth; sPTL, spontaneous preterm labour.

The study cohort sizes ranged from 20 participants (Ref. 15) (Table 2) to 1118 participants (Ref. 46) (Table 2; Table S2).

Five studies reported different miRNAs as either increased or decreased in preterm cases, four non-targeted (Table 2) and one targeted study (Table S2) (Refs 15–18, 45). Sanders *et al*. (Ref. 17) highlighted six miRNAs that were significantly associated with shorter gestational length, including miR-142. Zhou *et al*. (Ref. 18) investigated miRNA transcripts related to EBF1 and determined that MIR4266, MIR1251, MIR601 and MIR3612 were associated with sPTB. Further research by Zhou *et al*. (Ref. 47) also identified the long non-coding RNAs, LINC00870 and LINC00094, in association with PTB.

Awasthi and Pandey (Ref. 46) demonstrated that TLR4 (Toll-like receptor 4) mRNA was increased in PTB cases in a targeted study. Interleukin 6 receptor (IL-6R) gene expression was identified in a non-targeted investigation by Lee *et al*. (Ref. 48) (Table S2).

### Proteomics biomarkers

A total of 70 targeted proteomic studies met the review criteria, followed by nine non-targeted research (Table 3).

**Table 3.** Non-targeted proteomics studies (*n* = 9) of PTB proteomics maternal biomarkers

Study	Proteins identified	Phenotype	Sample type	Total number of maternal participants	Technology/technique	*P* value
Kim *et al*. (Ref. 49)	**Up-regulated** proteins in PTB: ZNF185, TRIM33, IGHV4–39, TOLLIP, TF, S100A10, RPLP2, IMPA1, CP, METAP2, BST1, HSPA6, LRRFIP1, RAC1, A1BG, IGHV1–8, AGT **Down-regulated** proteins in PTB: VAMP8, SDCBP2, FCAR, CREG1, ACP1, GLUL, CLCA4, SERPINB7, S100P, DMBT1, DPP4, KRT14, SERPINB2, EPS8L2, DSTN, GNB1, TP53I3, ARSB, KRT17, TGM3, DCD, RPTN, APEX1, GDF15, CCT2, SYNCRIP, FLG2, CPM, KRT2, KRT9, SPRR2D, KRT10, FLG, KRT1, LCE3D	PTB (<37 weeks)	Cervicovaginal fluid	12 [PTB *n* = 5; control *n* = 7]	iTRAQ and 2D-nLC-MS/MS	*P* < 0.05
Hallingstrom *et al*. (Ref. 50)	**Increased protein expression:** lipocalin-15; microfibril-associated glycoprotein 4 **Decreased protein expression:** neutrophil gelatinase-associated lipocalin; plasminogen activator inhibitor 1; extracellular superoxide dismutase [Cu-Zn]; insulin-like growth factor-binding protein 5; insulin-like growth factor-binding protein 7; semaphorin-3B; urotensin-2	PTD (<37 weeks)	Amniotic fluid	59 [sPTB *n* = 22, control *n* = 37]	LC-MS and ELISA	*P* < 0.05
Te-Yao *et al*. (Ref. 51)	Apolipoprotein A-IV; lumican; kininogen-1; T-complex protein 1 subunit theta; Vitamin D-binding protein; serum albumin; SPARC	PTB (<37 weeks)	Amniotic fluid	57 [PTB *n* = 36, control *n* = 21]	2D-DIGE and MALDI TOF/TOF	*P* < 0.05
D'Silva *et al*. (Ref. 52)	Of 30 proteins, includes: alpha-1-antitrypsin, vitamin D binding protein, apolipoprotein A-1	sPTB (<37 weeks)	Serum	20 [sPTB *n* = 10, control *n* = 10]	2D electrophoresis and LC/MS/MS	*P* < 0.05
Gunko *et al*. (Ref. 53)	Insulin-like growth factor-binding protein 1; bikunin; MMP-8; IL-6; IL-7; fibrinopeptide B; transcription elongation factor S-II; retinol-binding protein-2; ribosomal protein S6 kinase alpha-3; pigment epithelium-derived factor; lipocalin-1	PTB (<37 weeks)	Serum	20 [PTB *n* = 10, control *n* = 10]	Mass-spec	*P* < 0.05
Lynch *et al*. (Ref. 54)	Complement factors B and H coagulation factors IX and IX ab	PTB (20–37 weeks)	Plasma	129 [PTB *n* = 41, control *n* = 88]	Protein assay	*P* < 0.01
Liong *et al*. (Ref. 55)	Annexin A3, cystatin A (dimer), FABP5, GGCT, IL1ra, M/NEI, SCCA-1, thioredoxin, VDBP	PPROM (<37 weeks)	Cervical vaginal fluid	15 [PPROM *n* = 5, control *n* = 10]	2DDIGE	*P* < 0.05
Pereira *et al*. (Ref. 56)	52 proteins identified	sPTB (<34 weeks) and PTL (>34 weeks)	Serum	110 [sPTB *n* = 48, PTL *n* = 62]	MALDI/TOF-MS and 2-dimensional LC/MS/MS	*P* < 0.05
Pereira *et al*. (Ref. 57)	Calgranulin B; serotransferrin precursor; calgranulin A; fatty acid-binding protein; alpha-1-antitrypsin precursor; profilin-1; annexin A3; haptoglobin precursor; glyceraldehyde-3-phosphate dehydrogenase; alpha-1-acid glycoprotein 1 precursor; thymosin beta-4; lymphocyte cytosolic protein 1; calgranulin C; vitamin D-binding protein precursor; heat-shock protein beta-1; desmoplakin	sPTB (16–37 weeks)	Cervical vaginal fluid	18 [PTL control *n* = 6, PTB *n* = 6, control *n* = 6]	LC/LC-MS/MS, MudPIT and fluorescence 2DDIGE	*P* < 0.05

2DDIGE, two-dimensional differential in-gel electrophoresis; 2D-nLC-MS/MS, two-dimensional nanoflow liquid chromatography-tandem mass spectrometry; iTRAQ, isobaric tags for relative and absolute quantitation; LC/MS/MS, liquid chromatography mass spectrometry/mass spectrometry; MALDI/TOF-MS, matrix-assisted laser desorption ionisation time-of-flight mass spectrometry; PPROM, preterm pre-labour rupture of membranes; PTB, preterm birth; sPTB, spontaneous preterm birth.

Study cohorts were relatively small compared with genomics and transcriptomics, except for Lynch *et al*. (Ref. 54) and Pereira *et al*. (Ref. 56) with over 100 participants for untargeted proteomics studies (Table 3).

Elevated levels of lipocalin-type prostaglandin D2 synthase were detected in cervicovaginal secretions, which was associated with an increased risk of PTB (Ref. 58) (Table S3). Further inflammatory-associated proteins were identified in proteomic investigations, such as ILs reported by Gunko *et al*. (Ref. 53) and Liong *et al*. (Ref. 55) associated with PTB and PPROM, respectively (Table 3).

Targeted findings are summarised in Table S3. Non-targeted studies of the maternal proteome profile indicated growth factors as biomarkers, which were also reported in targeted literature (Table 3; Table S3). Other inflammatory-related protein markers (e.g. chemokines, ILs and TNF) were also reported in genomics and transcriptomics studies. Pawelczyk *et al*. (Ref. 59) described that TLR4 induces ILs, which increase the level of prostaglandins and cause uterine smooth muscle contraction leading to induction of PTB.

### Metabolomics biomarkers

A total of 20 metabolomics studies, 13 untargeted (Table 4) and seven targeted (Table S4) met the review criteria.

**Table 4.** Non-targeted metabolomics PTB maternal biomarker studies (*n* = 13) reported in literature

Study	Metabolites identified	Phenotype	Sample type	Total number of maternal participants	Technology/technique	*P* value
Stelzer *et al*.(Ref. 60)	Include: cortisol isomer, 1-methylhypoxanthine, 17-OH pregnenolone sulphate, 4-aminohippuric acid, arabitol, xylitol, 5-hydroxytryptophan, N-lactoylphenylalanine, pregnenolone sulphate	sPTB (<37 weeks)	Blood	63 [SPTB *n* = 63]	LC-MS/MS	*P* < 0.05
Zhao *et al*. (Ref. 61)	In microvesicles, PS (glycerophosphoserines) (34:0), PS (O-42:0), PI (glycerophosphoinositols) (O-36:1), C24 (OH) sulphatide, and PE (glycerophosphoethanolamines) (O-33:0) were lower in PTB (AUC >0.8)	PTB (<37 weeks)	Plasma	176 [PTB *n* = 68, control *n* = 108]	LC-MS/MS	NA
Morillon *et al*. (Ref. 62)	**Lower levels in sPTB:** 12 phosphatidylcholines, 7 phosphatidylethanolamines, 1 phosphatidylinositol, 2 ceramides and 4 sphingomyelins **Higher level in sPTB:** diaglyceride – DG (34:4)	sPTB (<37 weeks)	Plasma	48 [sPTB *n* = 16, control *n* = 32]	LC-MS	*P* < 0.05
Souza *et al*. (Ref. 63)	Undecane, dodecane and decane	PTB (<34 weeks)	Serum	321 [PTB *n* = 211, control *n* = 110]	GC-MS	*P* < 0.05
Considine *et al*. (Ref. 64)	Bile acids, prostaglandins, vitamin D and fatty acids	PTB (<37 weeks)	Serum	105 [PTB *n* = 50, control *n* = 55]	LC-MS	*P* < 0.01
Virgiliou *et al*. (Ref. 24)	**Serum lipids:** acylglycerophosphoserines, diacylglycerophosphoethanolamines, phosphatidylinositol, phosphatidylglycerol **Amniotic fluid:** glutamine, pyruvate, and inositol (lower in PTD), glutamate (higher in PTD). **Serum:** pyroglutamic acid (higher), hypoxanthine and tryptophan (lower in PTD)	PTD (<37 weeks)	Serum and amniotic fluid	70 [PTB *n* = 35, control *n* = 35]	UHPLC-MS	*P* < 0.01
Amabebe *et al*. (Ref. 25)	Acetate	PTB (<37 weeks)	Cervical vaginal fluid	277 [PTB *n* = 83, asymptomatic PTB *n* = 129, symptoms of PTL *n* = 65]	NMR spectroscopy	*P* = 0.003
Thomas *et al*. (Ref. 65)	NA	sPTB (<37 weeks)	Cervical vaginal fluid	60 [sPTB *n* = 30, control *n* = 30]	GC-MS	
Ghartey *et al*. (Ref. 66)	Metabolic pathways with observed changes: amino acids; peptide; carbohydrate; energy; lipid; nucleotide; cofactors and vitamins; xenobiotics	sPTB (<37 weeks)	Cervical vaginal fluid	20 [PTB *n* = 10, control *n* = 10]	LC-MS	NA
Maitre *et al*. (Ref. 67)	Steroid conjugate (*P* = 0.045) Lysine (*P* = 0.016) Trimethylamine-*N*-oxide (*P* = 0.032) Glycine (*P* = 0.049) *N*-Methyl-2-pyridone-5-carboxamide (*P* = 0.049) Formate (*P* = 0.009)	sPTB (<37 weeks)	Urine	464 [sPTB *n* = 88, control *n* = 275]	Proton NMR	
Graca *et al*. (Ref. 68)	Histidine, isoleucine/leucine, methionine, phenylalanine, valine	PTD (<37 weeks)	Amniotic fluid and urine	17	Ultra-high performance liquid chromatography -mass spectrometry (UHPLC−MS)	NA
Diaz *et al*. (Ref. 69)	Urine metabolites: 2-hydroxyisobutyrate (1.02 × 10^−2^) Choline (free) (1.51 × 10^−2^) Unknown metabolite (3.14 × 10^−2^)	Pre-premature delivery (PTD) (<37 weeks)	Urine and plasma	198	NMR	
Romero *et al*. (Ref. 37)	**Increased in PTL:** hexose cluster 6, dulcitol, urocanic acid, possible *N*-acetyl glutamine, 1-methyladenine, butanoic acid, beta hydroxyphenylethylamine, vitamin B6, salicylamide, oleic acid and other unknown metabolites **Decreased in PTL:** alanine, pyroglutamic acid, proline, glycine, glutamine, galactose, hexose cluster 5, hexose cluster 3, mannose, inositol, urea, 3-hydroxybutanoic acid, palmitate, octadecanoic acid and Butanedioic acid	PTL (<37 weeks)	Amniotic fluid	168	Gas and liquid chromatography and mass spectrometry	NA

GC-MS, gas chromatography–mass spectrometry; LC-MS, liquid chromatography–mass spectrometry; NMR, nuclear magnetic resonance; PPROM, preterm pre-labour rupture of membranes; PTB, preterm birth; PTD, preterm delivery; PTL, preterm labour; sPTB, spontaneous preterm birth; UHPLC-MS, ultra-high-performance liquid chromatography coupled to mass spectrometry.

The study sample sizes for metabolomics studies were overall higher than proteomics (Table 4; Table S4).

Most studies applied mass spectrometry followed by NMR approach on largely non-invasive samples including urine, amniotic fluid, cervicovaginal fluid and serum. Higher levels of metabolites such as glutamate were detected in PTB women, whereas glutamine, pyruvate and inositol were lower in PTB (Ref. 24) (Table 4). Virgiliou *et al*. (Ref. 24) also observed many significant uncharacterised saturated lipids. Both Considine *et al*. (Ref. 64) and Eick *et al*. (Ref. 70) (Table 4; Table S4) determined prostaglandins as a potential biomarker for PTB. Targeted investigations determined significant differences in nitrate oxide levels between preterm and term births (Table S4).

### Multi-omic studies

**Fig. 2.**
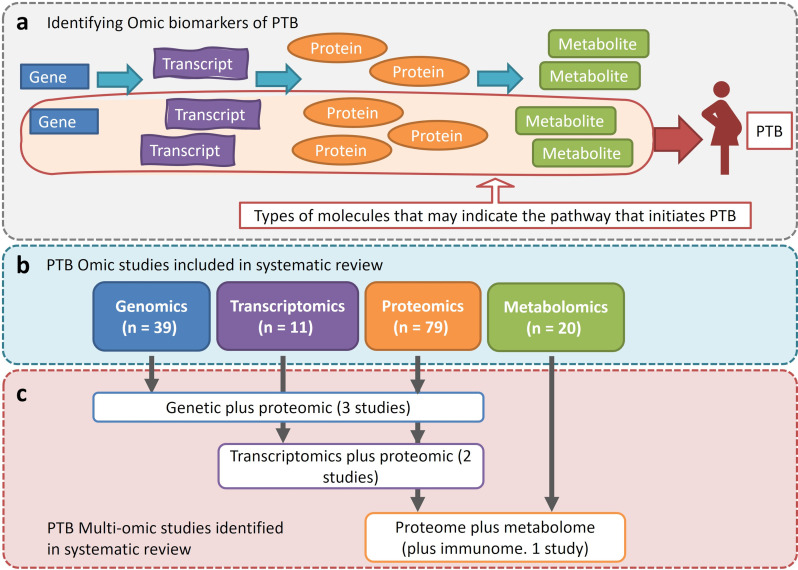
(a) Schematic diagram of omic data types in the direction of transcription and translation into end-products (such as proteins or metabolites). Of the vast number of omic molecules in the human system, some of these can be identified as part of the pathway (or pathways) that leads to the onset of PTB (as circled in red). These biomarkers can be detected using single or multiple omic investigations. (b) PTB omic studies identified and included, per type of omic data, in this systematic review. (c) From the identified PTB omic studies, the number of multi-omic studies per type of omic data is summarised.

As demonstrated in Figure 2a, multi-omic studies can identify different types of molecules that may be related to the same pathway or pathways involved in PTB. Across the individual omic literature searches, six studies explored more than one omic dataset and met the inclusion criteria for this systematic review (Fig. 2b and c; Table 5). Table 5 highlights which studies explored different omic datasets. Of these studies, the majority of them explored both genetic and proteomic data (Fig. 2c; Table 5).

**Table 5.** Summary of PTB studies that investigated more than one omic dataset identified in the systematic literature search (*N* = 6)

Study	Biomarker(s)	Omic data investigated
Stelzer *et al*. (Ref. 60)	Top markers included: 11 metabolomics, 17 proteomics, 14 immunome	Metabolome, proteome and immunome
Pandey *et al*. (Ref. 71)	*IL-6* polymorphisms and MMP-9 levels	Genetics and proteomics
Hao *et al*. (Ref. 72)	SNPs: *SOD2*, *SOD3* and *CAT*	Genetics and proteomics
Awasthi and Pandey (Ref. 46)	TLR4 mRNA and *TNF-α* genotype	Transcriptomics and genetics
Wang *et al*. (Ref. 73)	Mannose-binding lectin	Genetics and proteomics
Mustafa *et al*. (Ref. 74)	Organochlorine pesticides	Transcriptomics and proteomics

### Gestation threshold distribution

Each omic study utilised different gestational cut-offs to define PTB (or the subclinical groups of sPTB and PPROM). Comparison of these studies highlighted 29 weeks of gestation as the lowest time point applied, whereas the highest and most common was 37 weeks of gestation threshold (Fig. S1).

### Pathway analysis of biomarkers

Gene symbols (*n* = 14), miRNA names (*n* = 35), UniProt ID for proteins (*n* = 158) and KEGG IDs for metabolites (*n* = 60) were retrieved from the respective non-targeted omic studies and included in the pathway analyses. In total, 24 pathways were detected for genomics, 4 for transcriptomics, 102 for proteomics and 72 metabolomics, at *P* < 0.05. After correction for multiple testing (FDR *P* < 0.05), the number of significant pathways reduced to 2 for transcriptomics, 44 for proteomics and 19 for metabolomic markers. No pathways were significant for genomics biomarkers at FDR *P* < 0.05. Top pathways for each omic discipline are summarised in Table S5.

Multiple insulin-like growth factor-binding proteins and ILs identified from the literature were indicated in the significant insulin-like growth factor-binding pathway (FDR *P* < 0.05) (Table S5). Other identified proteomic biomarkers such as pregnancy associated plasma protein A (PAPPA) were also mapped to this pathway, indicating the role of PAPPA in regulating insulin-like growth factor.

Solute-carrier transporter pathway (also significant at FDR *P* < 0.05) was obtained from the metabolomics biomarkers reported in PTB literature. This pathway matched with several input metabolites including acetate, creatinine and histidine.

Non-targeted transcriptomics pathway over-representation analysis highlighted cholesterol transport regulation pathway (FDR *P* < 0.05) (Table S5), in which ATP-binding cassette subfamily A type 1 (ABCA1) and G type 1 (ABCG1) were found.

The secretin family receptor pathway, involving G-protein-coupled receptors, mapped to the input genes calcitonin receptor (*CALCR*) and *WNT4*. These were related to female reproductive system development and *WNT4* was identified as a potential PTB biomarker by Zhang *et al*. (Ref. 10).

## Discussion

Many PTB biomarker studies (*N* = 149) were identified across genomics, transcriptomics, proteomics and metabolomics. Infection pathway-related molecules, for example IL-6, TNF, TLR4 and prostaglandins were commonly reported across these studies. Cytokines have been suggested to play a key role in PTB (Refs 42, 43, 48). Targeted proteomics literature also supports this (Table S3). Non-targeted proteomics studies reported a range of proteins involved in immunological pathways, for example apolipoproteins (Table 3). Considine *et al*. (Ref. 64), Eick *et al*. (Ref. 70) and Kumar *et al*. (Ref. 58) studied protein concentration of prostaglandins in association with PTB. Nuclear factor-*κβ* is a regulator of inflammation pathways that could be activated by TLR, TNF and ILs, initiating inflammatory and hence leading to early labour, as reviewed by MacIntyre *et al*. (Ref. 75).

Suggested genetic biomarkers, such as *TIMP2*, *COL4A3* and *TNF* variants, were identified in a similar pathway as *EBF1*, which is involved in the regulation of B-cells (Ref. 10). Furthermore, a follow-up study of these findings by Zhou *et al*. (Ref. 18) found that *EBF1*-related LINC00870 and LINC00094 were correlated to sPTB. No epigenetics studies met the review criteria, though the role of detected H3K4me3 was discussed in a genomics study by Zhang *et al*. (Ref. 10). Rappoport *et al*. (Ref. 12) is one of several studies that have hypothesised the association of foetal genes with onset of early labour. However, this review focussed on maternal biomarkers as a potential screening tool (preferably non-invasive) that could be implemented with enough time to provide medical intervention (Ref. 76).

Transcriptomics was identified as an increasingly popular omic field as multiple miRNAs were indicated as candidate biomarkers of PTB (Refs 15–18, 45). Notably, gene expression studies indicated that miR1253 was under-expressed in women with sPTB (Ref. 16) or increased expression of miR-142 was associated with shorter gestation (Ref. 17).

Proteomics was the most popular omic field, reporting the highest number of candidate biomarkers of PTB. Targeted proteomic biomarker studies were traditionally applied in PTB research; hence many candidate markers were highlighted in this review (Table S3). This overlapped with the other omic results, particularly for protein levels of TNF and ILs (Table S3; Table 3).

More recent studies of metabolomic markers have applied non-targeted approaches to detect multiple metabolite markers (Table 4), whereas earlier studies applied targeted assay for example, to detect nitric oxide metabolite levels (Ref. 77). Nitric oxide (which is involved in many physiological processes) was often explored because of its potential interactions with inflammatory pathway components, for example cytokines. Non-targeted methods, such as NMR, provide individual level data, which can be integrated with other omic data for personalised medicine. Six research articles that studied more than one omic field met the review criteria (Table 5; Fig. 2c). Figure 2a outlines how different types of omic molecules can correspond to the pathway (e.g. an inflammation-activated pathway) that initiates early labour. Though biomarkers can be detected by single omic studies, the interrelationship between these molecules cannot be inferred. Multi-omic studies offer a holistic ‘systems biology’ approach to understanding the ‘cross-talk’ between different omic molecules and determine the pathway leading to PTB (Ref. 78). However, multi-omic studies come with many challenges related to experimental design, sample preparation as well as handling and analysing large datasets (Ref. 78). Advanced computational skills and methods from field such as advanced statistics and machine learning (from artificial intelligence) are being applied across different medical research fields (Ref. 78).

Pathway analysis of non-targeted individual omic layers indicated cellular processes associated with infection-related functions. Few pathways were significant at FDR *P* < 0.05, possibly because of the low number of studies included in the analyses (Table S5). To date, no online platforms can implement pathway analysis of all four omic disciplines (genomics, transcriptomics, proteomics and metabolomics) using the molecule identifiers selected in this review, thereby, reducing the capability of investigating the interaction between the omic layers.

Meta-analysis including all the studies in this review could not be performed because of heterogeneity caused by differences in factors including technology or methods, study design, sampling time and PTB outcome definition. Samples were obtained from first trimester up to a few days before the patient delivered or started experiencing contractions prior to or around third trimester. Furthermore, different gestational cut-offs were applied across the studies (Fig. S1). Different ethnic populations were included in the genomics studies, which would cause genetic heterogeneity if meta-analyses were to be performed. Targeted and non-targeted studies could not be combined as this would lead to bias in interpretation and therefore, meta-analysis was not suitable for this review.

The majority of studies applied <37 weeks cut-off to define PTB (Fig. S1), though this is considered late-PTB by the definition provided by the WHO and thereby more likely to detect molecular changes closer to term delivery. Fewer studies applied earlier gestational age thresholds; the earliest was 29 weeks as reported by Annells *et al*. (Ref. 38). Each study also collected different types of samples at differing gestational timepoints, for example, serum at 11–13 weeks of gestation by Kansu-Celik *et al*. (Ref. 79) or plasma at 16–20 weeks of gestation by Ellis *et al*. (Ref. 80). These studies explored the molecular characteristics of PTB sub-clinical phenotypes sPTB or PPROM, however this increased the clinical heterogeneity in this review as other studies did not distinguish between the PTB phenotypes. Most likely all clinical sub-groups were grouped under the term ‘PTB’ or ‘sPTB’ using the <37 weeks gestation cut-off. This raises another issue that there is no standard classification system of PTB phenotypes, as described by Goldenberg *et al*. (Ref. 9). Any combination of risk factors can affect women of different ethnic and socio-economic status at varying gestational timepoints (Ref. 9). Women recruited at the time of onset of contractions or admission to hospital because of symptoms of early labour were excluded from the review as labour had already started. This would not be an ideal predictive biomarker, especially for asymptomatic women.

Current PTB prevention guidelines in the UK recommend that women experiencing PTB symptoms at ≤34 weeks of gestation are in most need of PTB prevention treatment, therefore a diagnostic screen before this timepoint would be appropriate (Ref. 81). An ideal biomarker for PTB would be implemented in early stages of pregnancy and utilise non-invasive sampling techniques (for instance, systemic fluids, including maternal blood samples (serum or plasma samples) or amniotic fluid collected from the vagina), similar to the studies highlighted in this review. This would also enable clinical decisions to be made earlier that could improve pregnancy outcomes (Ref. 76). The biomarker should be technically simple to execute in non-specialist clinical settings (such as laboratories), low cost, high accuracy and reasonably quick to obtain results. The integration of this type of screening into the healthcare system and acceptability amongst the pregnant population would also need to be considered. Few prenatal diagnostic tests are offered, and these are often to detect severe conditions affecting the foetus, which raises ethical implications for mothers, as would be the case for PTB screening. In addition to this, the biomarker (or a panel of biomarkers) should be suitable for a multi-ethnic population. However, further work would be required to ensure the validity of the biomarker, which requires large, prospective, multicentre multi-omic studies. One of the current efforts include the ‘March of Dimes’, an organisation based in the USA (https://www.marchofdimes.org/index.aspx), who have funded several international PTB research centres that are leading global multi-centre PTB omic research. Large cohort studies, with a robust study design, can enable firm conclusions to be drawn.

The studies outlined in this review implemented different experimental approaches and study designs for biomarker identification, but these were not validated, or the diagnostic value was not reported. This limitation results from the lack of standard for reporting omic biomarkers and quality assessment of omic studies (Ref. 82). Future work could involve development of a quality assessment tool for the articles that applied a specific study design and reported biomarker diagnostic value and by adapting current guidelines.

The literature has demonstrated that multi-omic studies can be completed on the same cohort of women. Integration of clinical data with omic biomarkers could lead to predictive models of pregnancy outcome. Our experiences of prospectively recruiting women to a multi-omic study of PTB highlighted the need for a large sample size to retain statistical power at the multi-omic level, whereby samples loss occurred within the individual omic analyses then during multi-omic data integration (Refs 83, 84). Study participant recruitment and sample collection during pregnancy required trained staff and is time-intensive, despite this, participants missing appointments could not be avoided leading to further data loss. The number of PTB cases is relatively low in a single population and even lower when categorised into the sub-groups sPTB and PPROM (Refs 83, 84). This reiterates the need for a multi-centre recruitment approach.

To conclude, this review has demonstrated that PTB omic studies have reported biomarkers in concordance with the key risk factor of infection or inflammation, hence suggesting the role of infection or immunological pathway components in initiating early labour. This also highlights the need for multi-omic studies to determine biomarkers and pathways associated with PTB based on interactions between omic layers.

## Supplementary material

For supplementary material accompanying this paper visit http://doi.org/10.1017/erm.2022.13. click here to view supplementary material
