# Supplementary material for: Systematic review of preterm birth multi-omic biomarker studies
Source: Expert Rev Mol Med. 2022 Apr 5;24:e18. doi: 10.1017/erm.2022.13 (PMC9884789; doi:10.1017/erm.2022.13)
Supplement: Supplementary file 1 [file S1462399422000138sup001.docx]

**Supporting Information**

Systematic review of preterm birth multi-omic biomarker studies

**Juhi K. Gupta**^1,2^* and Ana Alfirevic^1,2^

^1^ Wolfson Centre for Personalised Medicine, Department of Pharmacology and Therapeutics, Institute of Systems, Molecular and Integrative Biology, University of Liverpool, Liverpool, L69 3GL, UK

^2^ Harris-Wellbeing Research Centre, University Department, Liverpool Women's Hospital, Liverpool, L8 7SS, UK

**Table of contents**

Table S1: Targeted genomics studies.

Table S2: Targeted transcriptomics studies.

Table S3: Targeted proteomics studies.

Table S4: Targeted metabolomics studies.

Table S5. Omics pathway analysis results.

Figure S1. Gestation distribution in PTB omics biomarker literature.

Table S1. Targeted preterm birth genomics biomarker studies. Maternal genes/SNPs biomarkers identified in the literature (n=30).

| Study | Ethnic groups | Genes/SNPs identified | Phenotype | Total number of maternal participants | Technology/  technique | P value |
| --- | --- | --- | --- | --- | --- | --- |
| [Pandey et al. (2020)](#_ENREF_114)  (Ref. 1) | Indian | *MMP-1*_rs1799750  *MMP8*_rs11225395  *IL-6_*rs1800795  *MMP-8_*rs2155052  *MMP-9_*rs3918242 | PTB (<37 weeks) | 510  [PTB n=255, control n=255] | Restriction Fragment length polymorphism | NA |
| [Hao et al. (2020)](#_ENREF_55) (Ref. 2) | Chinese | *SOD2* (rs2758352), *SOD3* (rs699473), and *CAT* (rs769214) | SPTB (<37 weeks) | 528  [PTB n =147, control n=381] | MALDI-TOF MS (MassArray, Sequenom) | P < 0.01 |
| [Gillespie et al. (2017)](#_ENREF_47) (Ref. 3) | African American (non-Hispanic) | GG genotype of *IL1RN* SNP rs2637988 | Birth timing (28–32 weeks) | 89 | TaqMan SNP Genotyping Assays | P=0.04 |
| [Frey et al. (2016)](#_ENREF_42) (Ref. 4) | African American | **Alleles sPTB vs Control deliveries:**  *PRKCA* (rs7225452, rs4486944, rs6504424, rs16960070)  *FLT1* (rs12428494)  **Genotypes sPTB <37 weeks:**  *MMP2* (rs11639960)  *TIMP2* (rs2277698)  *IL16* (rs7171517)  *PRKCA* (rs16960070, rs7225452, rs6504424, rs4486944)  **sPTB before <34 weeks:**  *MMP1* (rs7945189)  *MMP2* (rs11639960)  *LIFR-AS1* (rs6451398) | sPTB  (<37 weeks and <34 weeks) | 833 | Customized 1536 SNP Illumina Golden Gate genotyping array | Findings reported: p<7.4x10^-5^ |
| [Langmia et al. (2016)](#_ENREF_83) (Ref. 5) | Malays, Chinese or Indians | *IL1R2* rs2072476 | PTB  (24-36 weeks) | 664 | MALDI-TOF MS (MassArray, Sequenom) | p=0.017 |
| [Ramos et al. (2016)](#_ENREF_130)  (Ref. 6) | Brazilian population:  European, Amerindian and African ancestries | *IL10-1082G* (rs1800896) and *TLR2A* (rs4696480) alleles increased risk for PPROM  *TNFA-238A* (rs361525) - protective against PTL | PTL with intact membranes (<37 weeks), PPROM | 603  [PTL n=136, PPROM n=65, control n=402] | TaqMan SNP Genotyping Assays | P< 0.05 |
| [Bream et al. (2013)](#_ENREF_16) (Ref. 7) | American and Danish | *ENPP1*, *IGFBP3*, *DHCR7* and *TRAF2*. | PTB  (<37 weeks) | 37 | TaqMan | P<0.01 |
| [Falah et al. (2013)](#_ENREF_37) (Ref. 8) | Discovery cohort, USA: white, African-American, Finnish and Hispanic.    Replication cohort: Danish. | **SNPs in US white mothers:**  *IL23R* (rs11465804); *LPL* (rs12678919);  *CDKN2* (rs4977574); *MHC* (rs6457617);  *HLA-DRB1* (rs6457620); *HLA-DRA* (rs9268645); *HLA-DQA1* (rs9272346)  **US African-American mothers:**  *PCSK9* (rs11591147); *GCKR* (rs780094);  *FGB/FGA/FGG* (rs6056)  **US Hispanic mothers:**  *LDLR* (rs6511720)  **Danish, PTB protective association:**  *HLA-DQB1* (rs1063355) | PTB (<37 weeks) | Discovery cohort: 1,792  [sPTB n=673, control n=1,119]  Replication cohort: 2,000  [PTB n=1,000, control n=1,000] | MALDI-TOF MS (MassArray, Sequenom) | P<0.05 |
| [Harmon et al. (2013)](#_ENREF_56)  (Ref. 9) | European-American, African-American | **European:**  *IFNGR2* – strong association  *KIR3DL2* – strong association  *IL4* and *IL13* (rs3091307)  **European and African Americans:**  reduced risk of PTB - *IL12A* and *CSF2* | sPTB  (<37 weeks) | 1,646  [PTB n=347, control n=1299] | Illumina GoldenGate SNP assay | NA |
| [Iwanaga et al. (2011)](#_ENREF_65)  (Ref. 10) | Japanese | FcγRIIB-nt645 + 25AA carriers had a significantly shorter gestational period and a higher rate of PTB (periodontitis) | PTB  (<37 weeks) | 122  [PTB n=51, control n=71] | Big Dye Cycle Sequencing (Applied Biosystems) | p=0.032 |
| [Romero et al. (2010)](#_ENREF_134)  (Ref. 11) | Hispanic origin | **GWAS analysis:**  *TIMP2* (rs2277698)  **Global haplotype analysis**:  *COL4A3* (rs1882435-rs10178458-rs55997063) | sPTB  (<37 weeks) | 822  [PTB n=223, control n=599] | MALDI-TOF MS (MassArray, Sequenom) | P<0.01 |
| [Mustafa et al. (2010)](#_ENREF_107) (Ref. 12) | Indian | *GSTM1-/GSTT1-* (increased risk of PTL) | PTL  (<37 weeks) | 123  [PTB n=60, control n=63] | Multiplex PCR | p=0.028 |
| [Romero et al. (2010)](#_ENREF_132)  (Ref. 13) | Hispanic origin | *TIMP2* (rs2277698); *ANG* (rs11701); *TLR1* (rs3923647); *NOS3* (rs3730305); *COL4A3* (rs1882435); *PTGER1* (rs3745459) | PPROM  (PTB, 21-36 weeks) | 824  [pPROM n=225, control n= 599] | MALDI-TOF MS (MassArray, Sequenom) | P<0.01 |
| [Kwon et al. (2009)](#_ENREF_82) (Ref. 14) | Korea | *ICAM-1* K469E polymorphism | sPTB  (<37 weeks) | 206  [PTB n=53, control n=153] | PCR and auto-sequencing | P<0.05 |
| [Gebhardt et al. (2009)](#_ENREF_43)  (Ref. 15) | South African (Black, European and Asian ancestry) | *LGALS13b*  *TNFA* | PTB  (<37 weeks) | 450 | PCR | P<0.05 |
| [Menon et al. (2008)](#_ENREF_98) (Ref. 16) | African Americans and Caucasians | *TNF-α,* *TNFR1* and *TNFR2* | PTB  (<37 weeks) | 606  [PTB n=221, control n=385] | Illumina GoldenGate genotyping | P<0.05 |
| [Velez et al. (2008)](#_ENREF_159) (Ref. 17) | Caucasian | *CRHBP* (rs1875999, rs32897, rs10055255); *FV* (rs9332624); *IL5* (rs739718); *tPA* (rs879293); *PTGER3* (rs977214, rs594454); *SCNN1A/sTNF-R1* (rs3764874) | PTB  (<37 weeks) | 339  [PTB n=145, control n=194] | Illumina's GoldenGate genotyping | P<0.01 |
| [Steffen et al. (2007)](#_ENREF_148)  (Ref. 18) | All ethnic groups | **Gestational age:**  *ABCA1* (rs4149313), *APOE* (rs7412), *LCAT* (rs1109166), *LIPC* (rs6083)  **Prematurity (PTB):**  *DHCR24* (rs2274941) | Gestational age (22 to 36 weeks) and PTB | 351 | TaqMan chemistry | P<0.05 |
| [Ehn et al. (2007)](#_ENREF_29) (Ref. 19) | American | Progesterone receptor (PGR):  rs653752, rs503362, rs4754732, rs1942836, alu insertion | PTB  (<37 weeks) | 415 | TaqMan | P < 0.05 |
| [Menon et al. (2006)](#_ENREF_99) (Ref. 20) | European-American | *TNF-α, IL-6, IL-6R* | sPTB  (<36 weeks) | 422  [PTB n=101, control n=321] | TaqMan assay | P<0.001 |
| [Kalish et al. (2006)](#_ENREF_70) (Ref. 21) | American (mixed) | **PPROM alleles:**  *CD14*T; CD14*T; IL1RN*2; CD14*T; hsp70-2*G* | PPROM and sPTB  (<37 weeks) | 110  [PPROM n=28, sPTB n=72, control n=10] | PCR | P<0.05 |
| [Kalish et al. (2005)](#_ENREF_69)  (Ref. 22) | American (mixed) | *TNFRSF6* (genotype)  *TNFRSF6*G* (allele) | PPROM  (37 weeks) | 119  [PPROM n=33, SPTB n=9, control n=77] | PCR | P<0.05 |
| [Annells et al. (2004)](#_ENREF_4)  (Ref. 23) | European | **PTB:**  *IL10 −1082A/−819T/−592A*,  *TNF +488A/−238G/−308G*,  *IL4 −509C/C*,  *MBL2* codon 54Asp  **PPROM:**  *IL10 −1082G/−819C/−592C* | PTB (<29 weeks) and PPROM | 387  [PTB n=202, control n=185] | PCR | P<0.05 |
| [Hartel et al. (2004)](#_ENREF_57) (Ref. 24) | Caucasian | *IL6-174G* | PTB  (<37 weeks) | 747  [PTB n=466, control n=281] | PCR | P=0.018 |
| [Valdez et al. (2004)](#_ENREF_158)  (Ref. 25) | Mexican | **Control vs Preterm:**  *MTHFR C677T*  *ACE D* (allele)  **Preterm without infection:**  *MTHFR T* polymorphism  **Premature rupture of membranes:**  *ACE* *D* | PTB (22–36 weeks), PROM and infection | 253  [PTB n=89, control n=164] | PCR | P<0.05 |
| [Miller et al. (2015)](#_ENREF_101) (Ref. 26) | Black, Hispanic, White, Other | *β2AR* or *ADRB2* (rs1042713, rs1042714) | Cervix length and PTB (<37 weeks) | 439 | Sanger sequencing and electrophoresis | NA |
| [Schmid et al. (2010)](#_ENREF_142) (Ref. 27) | Austria | *CRHR2* G/A (rs2267717) | PTB  (<37 weeks) | 200  [PTB n=100, control n=100] | PCR | NA |
| [Uvuz et al. (2009)](#_ENREF_156) (Ref. 28) | Turkish | *FVL, FVC, FII, MTHFR C677T, MTHFR C1298T, ACE* | PTB  (28-36 weeks) | 100  [PTB n=50, control n=50] | Nanogen Microarray | NA |
| [Wang et al. (2017)](#_ENREF_163) (Ref. 29) | Taiwanese | *MBL2* | PTB  (<37 weeks) | 306  [PTB n=51, control n=255] | TaqMan | NA |
| [Fortunato et al. (2008)](#_ENREF_41)  (Ref. 30) | Black and White women of non-Hispanic origin | *IL-6* (rs1554606, rs1880243, rs1800795, rs1800796, rs1800797)  *IL-6R* (rs4845622, rs4845623, rs6687726)  *TNF-α* (rs769178, rs1799964, rs1800629, rs1800683, rs3179004)  *TNFR1* (rs740841, rs1860545, rs2302350, rs3764874, rs4149577)  *TNFR2* (rs474247, rs590368, rs616645, rs653667, rs976881, rs1061631, rs5746053) | sPTB  (<36 weeks) | 1195  [PTB n=448, control n=747] | TaqMan assay | NA |

MALDI-TOF MS = matrix-assisted laser desorption/ionization time of flight mass spectrometry; PPROM = preterm pre-labour rupture of membranes; PTB = preterm birth;

PTL = preterm labour; sPTB = spontaneous preterm birth

Table S2. Preterm birth targeted transcriptomics studies of maternal samples (n=6).

| Study | Transcripts Identified | Phenotype | Sample type | Total number of maternal participants | Technology/  technique | P value |
| --- | --- | --- | --- | --- | --- | --- |
| Manuck et al. (2021)  (Ref. 31) | **Genes at < 34 weeks:**  TLR2; DUSP1; RUNX3; PPP3CA; B2M; TLR4; IL10RA; PREX1; CYC1  **Genes at < 37 weeks:**  TLR2; DUSP1; B2M; TLR4; PREX1; IL10RB; RUNX3; PPP3CA; CXCR3; DDAH2; IL10RA; HSP90AB1; NCF1; RNF7 | spontaneous PTB  (<37 weeks and <34 weeks) | Blood | 136  [PTB (n=68), control (n=68] | Custom NanoString mRNA panel | P < 0.05 |
| [Zhou et al. (2021)](#_ENREF_171) (Ref. 32) | LINC00870 and LINC00094 | sPTB  (<37 weeks) | Blood | 165  [sPTB (n = 51), control (n=114)] | Bioinfomatics analysis using public databases | P < 0.05 |
| [Zhou et al. (2020)](#_ENREF_172) (Ref. 33) | EBF1-based miRNA transcripts (*MIR4266, MIR1251, MIR601,*  *MIR3612*) | sPTB  (<37 weeks) | Blood | 157  [sPTB n=51, control n=106] | Bioinformatics tools used: miRWalk and STarMirDB | P ≤ 0.018 |
| [Awasthi and Pandey (2019)](#_ENREF_6)  (Ref. 34) | TLR4 mRNA (increased expression in cases) | PTB  (<37 weeks) | Blood | 1118  [PTB n=559, control n=559] | Real-time PCR | P<0.001 |
| [Mustafa et al. (2015)](#_ENREF_106)  (Ref. 35) | COX-2 (increase fold change)  MnSOD and CAT (decreased fold change) | sPTB  (<37 weeks) | Blood | 100  [sPTB n=50, control n=50] | RT-PCR | P < 0.05 |
| [Lee et al. (2011)](#_ENREF_85) (Ref. 36) | IL-6R | PPROM and PTL (<37 weeks) | Amniotic fluid | 301  [Of which: PTL n=131, PPROM n=91] | Quantitative real-time RT-PCR | P < 0.01 |

PPROM = preterm pre-labour rupture of membranes; PTB = preterm birth; PTL = preterm labour; sPTB = spontaneous preterm birth

Table S3. Summary of 70 preterm birth targeted proteomics biomarker studies identified in a systematic search using PubMed, Web of Science and Science Direct. Multiple research studies targeted same or similar proteins.

| Study | Protein | Gene symbol | UniProt ID |
| --- | --- | --- | --- |
| Afzal et al. (2017) (Ref. 37) | C-reactive protein | CRP | P02741 |
| Bakalis et al. (2012) (Ref. 38) | C-reactive protein | CRP | P02741 |
| Berkowitz et al. (1996) (Ref. 39) | Corticotropin-releasing hormone or factor (CRH, CRF or Corticoliberin) | CRH | P06850 |
| Bernstein et al. (1998) (Ref. 40) | Free beta-human chorionic gonadotropin (beta-hCG) fbhCG (Choriogonadotropin subunit beta 3) | CGB3 | P0DN86 |
| Beta et al. (2011)  (Ref. 41) | Alpha-fetoprotein (AFP) | AFP | P02771 |
| Biggio et al. (2005) (Ref. 42) | Matrix metalloproteinase-8 | MMP8 | P22894 |
| Cantonwine et al. (2016) (Ref. 43) | Apolipoprotein M (APOM) | APOM | O95445 |
|  | Inter-alpha-trypsin inhibitor heavy chain 4 (ITIH4) | ITIH4 | Q14624 |
|  | Kininogen-1 (KNG1) | KNG1 | P01042 |
|  | Phosphatidylcholine-sterol acyltransferase | LCAT | P04180 |
|  | Fibulin-1 | FBLN1 | P23142 |
|  | Inter-alpha-trypsin inhibitor heavy chain H2 | ITIH2 | P19823 |
|  | Alpha-1-antichymotrypsin | AACT | P01011 |
|  | Plasma kallikrein | KLKB1 | P03952 |
|  | Trypsin-3 | PRSS3 | P35030 |
|  | Coagulation factor XIII B chain | F13B | P05160 |
|  | Apolipoprotein L1 | APOL1 | O14791 |
|  | N-acetylmuramoyl-L-alanine amidase | PGRP2 | Q96PD5 |
|  | Thyroxine-binding globulin | SERPINA7 | P05543 |
|  | CD5 antigen-like | CD5L | O43866 |
|  | Carboxypeptidase N catalytic chain | CBPN | P15169 |
|  | Vitamin D-binding protein | VTDB | P02774 |
|  | Protein AMBP | AMBP | P02760 |
|  | Complement component C8 alpha chain | CO8A | P07357 |
|  | Inter-alpha-trypsin inhibitor heavy chain H1 | ITIH1 | P19827 |
|  | Transthyretin | TTR | P02766 |
|  | Coagulation factor XIII A chain | F13A1 | P00488 |
|  | Apolipoprotein A-I | APOA1 | P02647 |
|  | Magnesium transporter MRS2 homolog, mitochondrial | HPT | Q9HD23 |
| Catov et al. (2014) (Ref. 44) | nonesterified (free) fatty acids (NEFAs) * |  |  |
| Curry et al. (2009) (Ref. 45) | Interleukin 2 (IL-2) | IL2 | P60568 |
|  | Interleukin 6 (IL-6) | IL6 | P05231 |
|  | Tumour necrosis factor alpha (TNF-a) | TNF | P01375 |
|  | Interferon (IFN) gamma | IFNG | P63309 |
|  | Granulocyte colony-stimulating factor (GCSF) or macrophage (GM-CSF) | CSF2 | P04141 |
| Curry et al. (2007) (Ref. 46) | Interleukin 2 (IL-2) | IL2 | P60568 |
|  | Interleukin 6 (IL-6) | IL6 | P05231 |
|  | Tumour necrosis factor alpha (TNF-a) | TNF | P01375 |
|  | Interferon (IFN) gamma | IFNG | P63309 |
|  | Granulocyte colony-stimulating factor (GCSF) or macrophage (GM-CSF) | CSF2 | P04141 |
| Dane et al. (2013) (Ref. 47) | Pregnancy associated plasma protein (PAPP-A or Pappalysin-1) | PAPPA | Q13219 |
|  | Free beta-human chorionic gonadotropin (beta-hCG) fbhCG (Choriogonadotropin subunit beta 3) | CGB3 | P0DN86 |
| Dunn et al. (2019) (Ref. 48) | Complement component C3 | C3 | P01024 |
|  | Complement factor B | CFB | P00751 |
| Ellis et al. (2002)  (Ref. 49) | Corticotropin-releasing hormone (CRH or Corticoliberin) | CRH | P06850 |
| Esplin et al. (2011) (Ref. 50) | Other peptides * |  |  |
|  | Placental growth factor (PlGF) | PGF | P49763 |
|  | Corticotropin-releasing hormone (CRH or Corticoliberin) | CRH | P06850 |
|  | Ferritin | FTL | P02792 |
|  | Tumour necrosis factor alpha (TNF-a) | TNF | P01375 |
| Ferguson et al. (2014) (Ref. 51) | Interleukin 1 beta (IL-1β) | IL1B | P01584 |
|  | Interleukin 6 (IL-6) | IL6 | P05231 |
|  | Interleukin 10 (IL-10) | IL10 | P22301 |
|  | Tumour necrosis factor alpha (TNF-a) | TNF | P01375 |
|  | C-reactive protein | CRP | P02741 |
| Forest et al. (1996) (Ref. 52) | Albumin | ALB | P02768 |
| Hallingstrom et al. (2020) (Ref. 53) | Adiponectin | ADIPOQ | Q15848 |
|  | Brain-derived neurotrophic factor | BDNF | P23560 |
|  | C-C motif chemokine 2 (monocyte chemotactic protein 1) | CCL2 or MCP1 | P13500 |
|  | C-C motif chemokine 3 (macrophage inflammatory protein 1-a (MIP-1-a)) | CCL3 | P10147 |
|  | C-C motif chemokine 5 | CCL5 | P13501 |
|  | C-reactive protein | CRP | P02741 |
|  | Granulocyte colony-stimulating factor (GCSF) or macrophage (GM-CSF) | CSF2 | P04141 |
|  | Insulin-like growth factor-binding protein 1 (IGFBP-1) | IGFBP1 | P08833 |
|  | Insulin-like growth factor-binding protein 3 (IGFBP-3) | IGFBP3 | P17936 |
|  | Interferon (IFN) gamma | IFNG | P63309 |
|  | Interleukin 1 beta (IL-1β) | IL1B | P01584 |
|  | Interleukin 10 (IL-10) | IL10 | P22301 |
|  | Interleukin 6 (IL-6) | IL6 | P05231 |
|  | Interleukin 8 (IL-8) | CXCL8 | P10145 |
|  | interleukin-12A | IL12A | P29459 |
|  | Interleukin-17 | IL17 | Q5QEX9 |
|  | Interleukin-18 | IL-18 | Q9NQ49 |
|  | Leptin | LEP | P41159 |
|  | Macrophage migration inhibitory factor (MIF) | MIF | P14174 |
|  | Matrix metalloproteinase-9 | MMP9 | P14780 |
|  | Neurotrophin-3 | NTF3 | P20783 |
|  | Triggering receptor expressed on myeloid cells 1 | TREM1 | Q9NP99 |
|  | Tumour necrosis factor alpha (TNF-a) | TNF | P01375 |
|  | Tumour necrosis factor receptor 1 (TNFR1) | TNFR1 | P19438 |
| Hallingstrom et al. (2016) (Ref. 54) | C-reactive protein | CRP | P02741 |
|  | Triosephosphate isomerase | TPI1 | P60174 |
|  | Calcium-activated chloride channel regulator | CLCA1 | A8K7I4 |
|  | Malate dehydrogenase, cytoplasmic | MDH1 | P40925 |
|  | Cystatin-SN | CST1 | P01037 |
|  | Hemoglobin subunit delta | HBD | P02042 |
|  | Hemoglobin subunit alpha | HBA1 | P69905 |
|  | Hemoglobin subunit beta | HBB | P68871 |
|  | Glycodelin | PAEP | P09466 |
|  | Cathelicidin antimicrobial peptide | CAMP | P49913 |
| Holzman et al. (2001) (Ref. 55) | Corticotropin-releasing hormone (CRH or Corticoliberin) | CRH | P06850 |
| Holzman et al. (2013) (Ref. 56) | Corticotropin-releasing hormone (CRH or Corticoliberin) | CRH | P06850 |
|  | C-reactive protein | CRP | P02741 |
|  | Interleukin 1 beta (IL-1β) | IL1B | P01584 |
|  | Interleukin 2 (IL-2) | IL2 | P60568 |
|  | Interleukin-4 | IL4 | P05112 |
|  | Interleukin 6 (IL-6) | IL6 | P05231 |
|  | interleukin-12A | IL12A | P29459 |
|  | Interferon (IFN) gamma | IFNG | P63309 |
|  | Transforming growth factor beta-1 | TGFB1 | P01137 |
| Hudic et al. (2016) (Ref. 57) | Progesterone-Induced Blocking Factor | PIBF1 | Q8WXW3 |
| Hunter et al. (2016) (Ref. 58) | C-C motif chemokine 2 (CCL2) | CCL2 | P13500 |
|  | C-C motif chemokine 3 (CCL3) | CCL3 | P10147 |
|  | C-C motif chemokine 4 (CCL4) | CCL4 | P13236 |
|  | Interleukin 6 (IL-6) | IL6 | P05231 |
|  | Interleukin 1 beta (IL-1β) | IL1B | P01584 |
|  | Interleukin 8 (IL-8) | CXCL8 | P10145 |
| Kaijomaa et al. (2016) (Ref. 59) | Pregnancy associated plasma protein (PAPP-A or Pappalysin-1) | PAPPA | Q13219 |
| Kalinka et al. (2005) (Ref. 60) | Interleukin-1 alpha | IL1A | P01583 |
|  | Interleukin 6 (IL-6) | IL6 | P05231 |
|  | Interleukin 1 beta (IL-1β) | IL1B | P01584 |
|  | Interleukin 8 (IL-8) | CXCL8 | P10145 |
| Kallioniemi et al. (2013) (Ref. 61) | Insulin-like growth factor-binding protein 1 (IGFBP-1) | IGFBP1 | P08833 |
| Kansu-Celik et al. (2019) (Ref. 62) | Advanced glycation end products (AEGs) * |  |  |
| Kesrouani et al. (2016) (Ref. 63) | C-reactive protein | CRP | P02741 |
|  | Interleukin 6 (IL-6) | IL6 | P05231 |
|  | Matrix metalloproteinase-9 | MMP9 | P14780 |
| Kim et al. (2013)  (Ref. 64) | Vascular endothelial growth factor (VEGF) | VEGF | P15692 |
|  | Interleukin 6 (IL-6) | IL6 | P05231 |
|  | Matrix metalloproteinase-8 | MMP8 | P22894 |
|  | C-reactive protein | CRP | P02741 |
| Kim et al. (2005)  (Ref. 65) | Free beta-human chorionic gonadotropin (beta-hCG) fbhCG (Choriogonadotropin subunit beta 3) | CGB3 | P0DN86 |
| Kirkegaard et al. (2011) (Ref. 66) | Free beta-human chorionic gonadotropin (beta-hCG) fbhCG (Choriogonadotropin subunit beta 3) | CGB3 | P0DN86 |
|  | Pregnancy associated plasma protein (PAPP-A or Pappalysin-1) | PAPPA | Q13219 |
| Kirkegaard et al. (2010) (Ref. 67) | Free beta-human chorionic gonadotropin (beta-hCG) fbhCG (Choriogonadotropin subunit beta 3) | CGB3 | P0DN86 |
|  | Pregnancy associated plasma protein (PAPP-A or Pappalysin-1) | PAPPA | Q13219 |
| Kramer et al. (2010) (Ref. 68) | C-reactive protein | CRP | P02741 |
|  | Interleukin 1 beta (IL-1β) | IL1B | P01584 |
|  | Interleukin 10 (IL-10) | IL10 | P22301 |
|  | Interleukin 6 (IL-6) | IL6 | P05231 |
|  | Interleukin 8 (IL-8) | CXCL8 | P10145 |
|  | interleukin-12A | IL12A | P29459 |
|  | Interleukin-17 | IL17 | Q5QEX9 |
|  | Interleukin-18 | IL-18 | Q9NQ49 |
|  | Interleukin-4 | IL4 | P05112 |
|  | Matrix metalloproteinase-9 | MMP9 | P14780 |
|  | Interleukin-5 | IL5 | P05113 |
|  | Macrophage migration inhibitory factor (MIF) | MIF | P14174 |
|  | Matrix metalloproteinase-9 | MMP9 | P14780 |
|  | Triggering receptor expressed on myeloid cells 1 | TREM1 | Q9NP99 |
|  | C-C motif chemokine 3 (macrophage inflammatory protein 1-a (MIP-1-a)) | CCL3 | P10147 |
|  | Interferon (IFN) gamma | IFNG | P63309 |
|  | Transforming growth factor beta-1 | TGFB | P01137 |
|  | C-C motif chemokine 4 | CCL4 or MIP1B | P13236 |
|  | Brain-derived neurotrophic factor | BDNF | P23560 |
|  | Neurotrophin-3 | NTF3 | P20783 |
|  | Neurotrophin-4 | NTF4 | P34130 |
|  | Granulocyte colony-stimulating factor (GCSF) or macrophage (GM-CSF) | CSF2 | P04141 |
| Kumar et al. (2015) (Ref. 69) | Lipocalin-type prostaglandin D2 synthase (L-PGDS) | PTGDS | P41222 |
| Lee et al. (2016)  (Ref. 70) | Vascular endothelial growth factor (VEGF) | VEGF | P15692 |
|  | Placental growth factor (PlGF) | PGF | P49763 |
|  | Vascular endothelial growth factor receptor 1 | FLT1 | P17948 |
| Malamitsi-Puchner et al. (2006) (Ref. 71) | Intercellular adhesion molecule-1 (sICAM-1) | sICAM-1 | Q99930 |
|  | Vascular cell adhesion molecule | VCAM1 | P19320 |
|  | Neutrophil elastase | ELANE | P08246 |
|  | Secretory leukocyte proteinase inhibitor (SLPI) | SLPI | P03973 |
| Manning et al. (2019) (Ref. 72) | Interleukin 8 (IL-8) | CXCL8 | P10145 |
|  | Interleukin 1 beta (IL-1β) | IL1B | P01584 |
|  | Elafin | PI3 | P19957 |
|  | Beta-defensin 1 | HBD1 | P60022 |
|  | Granulocyte colony-stimulating factor (GCSF) or macrophage (GM-CSF) | CSF2 | P04141 |
|  | C-C motif chemokine 2 (monocyte chemotactic protein 1) | CCL2 or MCP1 | P13500 |
|  | Interleukin 10 (IL-10) | IL10 | P22301 |
|  | Interleukin 6 (IL-6) | IL6 | P05231 |
|  | Interleukin-4 | IL4 | P05112 |
|  | interleukin-12A | IL12A | P29459 |
| Massaro et al. (2009) (Ref. 73) | Interleukin 6 (IL-6) | IL6 | P05231 |
| McDonald et al. (2015) (Ref. 74) | Complement component 5a (C5a) | C5 | P01031 |
|  | Vascular endothelial growth factor receptor 1 | FLT1 | P17948 |
|  | Intercellular adhesion molecule-1 (sICAM-1) | sICAM-1 | Q99930 |
|  | Interleukin-18-binding protein | IL18BP | O95998 |
|  | Chitinase-3-like protein 1 (CHI3L1) | CHI3L2 | P36222 |
|  | Endoglin | ENG | P17813 |
|  | Angiopoietin-related protein 3 (AngptL3) | ANGPTL3 | Q9Y5C1 |
| McElrath et al. (2019) (Ref. 75) | Inter-alpha-trypsin inhibitor heavy chain 4 (ITIH4) | ITIH4 | Q14624 |
|  | Inter-alpha-trypsin inhibitor heavy chain H2 | ITIH2 | P19823 |
|  | Phosphatidylcholine-sterol acyltransferase | LCAT | P04180 |
|  | Serotransferrin | TF | P02787 |
|  | Fibulin-1 | FBLN1 | P23142 |
| Mijal et al. (2012) (Ref. 76) | Vascular endothelial growth factor receptor 1 | FLT1 | P17948 |
|  | Endoglin | ENG | P17813 |
|  | Placental growth factor (PlGF) | PGF | P49763 |
| Moawad et al. (2002) (Ref. 77) | Alkaline phosphatase (ALP) | ALPL | P05186 |
|  | Alpha-fetoprotein (AFP) | AFP | P02771 |
|  | Corticotropin-releasing hormone or factor (CRH, CRF or Corticoliberin) | CRH | P06850 |
| Neggers et al. (2000 ) (Ref. 78) | Alpha-fetoprotein (AFP) | AFP | P02771 |
|  | Zinc levels * |  |  |
| Ong et al. (2000)  (Ref. 79) | Pregnancy associated plasma protein (PAPP-A or Pappalysin-1) | PAPPA | Q13219 |
|  | Free beta-human chorionic gonadotropin (beta-hCG) fbhCG (Choriogonadotropin subunit beta 3) | CGB3 | P0DN86 |
| Ozer et al. (2005)  (Ref. 80) | Pregnancy associated plasma protein (PAPP-A or Pappalysin-1) | PAPPA | Q13219 |
|  | C-reactive protein | CRP | P02741 |
| Ozgu-Erdinc et al. (2014) (Ref. 81) | Alkaline phosphatase (ALP) | ALPL | P05186 |
|  | Lactate dehydrogenase (LDH) | LDHA | P00338 |
|  | Ferritin | FTL | P02792 |
|  | Interleukin 6 (IL-6) | IL6 | P05231 |
|  | C-reactive protein | CRP | P02741 |
|  | Ceruloplasmin | CP | P00450 |
| Parry et al. (2020) (Ref. 82) | Fibronectin | FN1 | P02751 |
|  | Extracellular matrix protein 1 | ECM1 | Q16610 |
|  | Laminin subunit alpha-3 | LAMA3 | Q16787 |
|  | Calsyntenin-1 | CLSTN1 | O94985 |
| Parry et al. (2014) (Ref. 83) | Apolipoprotein M (APOM) | APOM | O95445 |
|  | Kininogen-1 (KNG1) | KNG1 | P01042 |
|  | Pregnancy zone protein | PZP | P20742 |
|  | Corticosteroid-binding globulin | SERPINA6 | P08185 |
|  | Prothrombin | F2 | P00734 |
|  | Retinol-binding protein 4 | RBP4 | P02753 |
|  | Hyaluronan-binding protein 2 | HABP2 | Q14520 |
|  | N-acetylmuramoyl-L-alanine amidase | PGLYRP2 | Q96PD5 |
|  | Ficolin-3 | FCN3 | O75636 |
|  | Carboxypeptidase N catalytic chain | CPN1 | P15169 |
|  | Pappalysin-1 | PAPPA | Q13219 |
| Pawelczyk et al. (2010) (Ref. 84) | Toll-like receptor 4 (TLR4) | TLR4 | O00206 |
| Pearce et al. (2010) (Ref. 85) | Corticotropin-releasing hormone (CRH or Corticoliberin) | CRH | P06850 |
|  | Macrophage migration inhibitory factor (MIF) | MIF | P14174 |
|  | Tumour necrosis factor alpha (TNF-a) | TNF | P01375 |
|  | C-reactive protein | CRP | P02741 |
|  | Interleukin 1 beta (IL-1β) | IL1B | P01584 |
|  | Interleukin 6 (IL-6) | IL6 | P05231 |
| Pihl et al. (2009)  (Ref. 86) | Pregnancy-specific beta-1-glycoprotein (SP1) | PSG1 | P11464 |
| Pitiphat et al. (2005) (Ref. 87) | C-reactive protein | CRP | P02741 |
| Puchner et al. (2011) (Ref. 88) | Interleukin 1 beta (IL-1β) | IL1B | P01584 |
|  | Interleukin 10 (IL-10) | IL10 | P22301 |
|  | Interleukin-18 | IL-18 | Q9NQ49 |
| Puchner et al. (2012) (Ref. 89) | Tumour necrosis factor alpha (TNF-a) | TNF | P01375 |
|  | Cytochrome C | CYCS | P99999 |
| Rahkonen et al. (2010) (Ref. 90) | Insulin-like growth factor-binding protein 1 (IGFBP-1) | IGFBP1 | P08833 |
| Rosen et al. (2001) (Ref. 91) | Thrombin–antithrombin (TAT) * |  |  |
| Ruiz et al. (2016)  (Ref. 92) | Corticotropin-releasing hormone (CRH or Corticoliberin) | CRH | P06850 |
| Ruiz et al. (2012)  (Ref. 93) | Interleukin-1 receptor antagonist protein | IL1RN | P18510 |
|  | Interleukin 6 (IL-6) | IL6 | P05231 |
|  | Interleukin 10 (IL-10) | IL10 | P22301 |
| Saade et al. (2016) (Ref. 94) | Insulin-like growth factor-binding protein 4 | IGFBP4 | P22692 |
|  | Sex hormone-binding globulin | SHBG | P04278 |
| Shin et al. (2016)  (Ref. 95) | Insulin-like growth factor-binding protein 1 (IGFBP-1) | IGFBP1 | P08833 |
|  | Insulin-like growth factor-binding protein 3 (IGFBP-3) | IGFBP3 | P17936 |
|  | Insulin-like growth factor I (IGF-1) | IGF1 | P05019 |
| Smith et al. (2002) (Ref. 96) | Pregnancy associated plasma protein (PAPP-A or Pappalysin-1) | PAPPA | Q13219 |
|  | Free beta-human chorionic gonadotropin (beta-hCG) fbhCG (Choriogonadotropin subunit beta 3) | CGB3 | P0DN86 |
| Soni et al. (2019)  (Ref. 97) | Free beta-human chorionic gonadotropin (beta-hCG) fbhCG (Choriogonadotropin subunit beta 3) | CGB3 | P0DN86 |
| Sorokin et al. (2010) (Ref. 98) | Matrix metalloproteinase-9 | MMP9 | P14780 |
|  | C-reactive protein | CRP | P02741 |
|  | Interleukin 6 (IL-6) | IL6 | P05231 |
| Straughen et al. (2012) (Ref. 99) | Vascular endothelial growth factor receptor 1 | FLT1 | P17948 |
| Tamura et al. (1996) (Ref. 100) | Zinc, Copper, iron * |  |  |
|  | Ferritin | FTL | P02792 |
|  | Transferrin (Serotransferrin) | TF | P02787 |
|  | Albumin | ALB | P02768 |
|  | C-reactive protein | CRP | P02741 |
|  | Alpha-2-macroglobulin | A2M | P01023 |
|  | Ceruloplasmin | CP | P00450 |
|  | Retinol-binding protein 1 | RBP1 | P09455 |
| Thorsen et al. (2001) (Ref. 101) | Interleukin 1 beta (IL-1β) | IL1B | P01584 |
|  | Tumour necrosis factor alpha (TNF-a) | TNF | P01375 |
|  | Interleukin 6 (IL-6) | IL6 | P05231 |
|  | Interleukin 10 (IL-10) | IL10 | P22301 |
| Vogel et al. (2006) (Ref. 102) | Prorelaxin H2 (or relaxin) | RLN2 | P04090 |
| Wallenstein et al. (2016) (Ref. 103) | Vascular endothelial growth factor receptor 1 | FLT1 | P17948 |
|  | Tumour necrosis factor receptor 1 (TNFR1) | TNFR1 | P19438 |
|  | Interleukin-2 receptor subunit alpha | IL2RA | P01589 |
| Whitcomb et al. (2009) (Ref. 104) | Granulocyte colony-stimulating factor (GCSF) or macrophage (GM-CSF) | CSF2 | P04141 |
| Zhang et al. (2017) (Ref. 105) | Complement decay-accelerating factor (CD55) | CD55 | P08174 |
|  | Integrin alpha-M | ITGAM or CD11B | P11215 |
|  | Hyaluronic acid receptor CD44 | CD44 | P16070 |
|  | C-X-C chemokine receptor type 1 | CXCR1 | P25024 |
|  | C-C chemokine receptor type 2 | CCR2 | P41597 |
| Zhu and Yang (2018) (Ref. 106) | C-reactive protein | CRP | P02741 |
|  | Interleukin 6 (IL-6) | IL6 | P05231 |
|  | Macrophage migration inhibitory factor (MIF) | MIF | P14174 |

*= no UniProt IDs were identified for nonesterified (free) fatty acids (NEFAs), iron, copper, zinc, thrombin-antithrombin (TAT) and advanced glycation end products (AGEs).

Table S4. Preterm birth metabolomics targeted methods reported in maternal biomarker discovery literature (n=7).

| Study | Metabolites Identified | Phenotype | Sample type | Total number of maternal participants | Technology/  technique | P value |
| --- | --- | --- | --- | --- | --- | --- |
| Patil et al. (2021)  (Ref. 107) | 11-deoxycorticosterone (DOC) | PTB (<32 weeks) | Serum | 93  [n= 28 < 32 weeks, n= 40 between 32 - 36 weeks and n=25 > 37 weeks] | UPLC/MS-MS | NA |
| [Eick et al. (2020)](#_ENREF_30)  (Ref. 108) | 8-iso-prostaglandin F2α (8-iso-PGF2α) and prostaglandin F2α (PGF2α) | PTB  (<37 weeks) | Urine | 469  [PTB n=50, control n=396] | stable isotype  dilution gas chromatography-negative ion chemical ionization-mass  spectrometry | P <0.05 |
| [Rosen et al. (2019)](#_ENREF_135)  (Ref. 109) | Free 8-iso-PGF2α (including its metabolite: 2,3-dinor-5,6-  dihydro-15-F2 t-isoprostanes) and PGF2α. | PTB  (<37 weeks) | Urine | 740  [PTB n=61, control n=679] | gas chromatography negative  ion chemical ionization-mass spectrometry | NA |
| [Ferguson et al. (2014)](#_ENREF_39)  (Ref. 110) | Phthalates | PTB  (<37 weeks) | Urine | 482  [PTB n=130, control n=352] | Solid-phase extraction (SPE) and mass spectrometry | NA |
| [Giannella et al. (2011)](#_ENREF_46)  (Ref. 111) | Nitric oxide metabolite (NOx) | PTL  (<34 weeks) | Cervical and gingival swabs | 820  [PTL n=400, control n=420] | Griess reaction | NA |
| [Adibi et al. (2009)](#_ENREF_1)  (Ref. 112) | Mono-2-ethylhexyl phthalate  Mono-2-ethyl-5-oxohexyl phthalate | PTB  (<37 weeks) | Urine | 441 | Solid-phase extraction (SPE) and mass spectrometry | NA |
| [Longini et al. (2007)](#_ENREF_87)  (Ref. 113) | F2-IP (Isoprostanes) | PPROM  (<37 weeks) | Amniotic fluid | 113  [PPROM n=16, no PPROM n=97] | Immunoassay | p<0.0001 |

UPLC/MS-MS = ultraperformance liquid chromatography-tandem mass spectrometry.

Table S5. Top significant pathways (FDR p<0.05, or p<0.05 for genomics) of biomarkers reported in PTB omics literature using Reactome pathway analysis tool (Refs 114, 115).

| Omics | Pathway identifier | Pathway name | Entities found | Entities p | Entities FDR |
| --- | --- | --- | --- | --- | --- |
| Genomics | R-HSA-373080 | Class B/2 (Secretin family receptors) | 2 | 0.003784 | 0.198288 |
| Transcriptomics | R-HSA-9029569 | NR1H3 & NR1H2 regulate gene expression linked to cholesterol transport and efflux | 5 | 2.47E-04 | 0.019023 |
| Proteomics | R-HSA-381426 | Regulation of Insulin-like Growth Factor (IGF) transport and uptake by Insulin-like Growth Factor Binding Proteins (IGFBPs) | 20 | 1.11E-16 | 7.18E-14 |
| Metabolomics | R-HSA-425407 | SLC-mediated transmembrane transport | 20 | 6.63E-07 | 5.02E-04 |


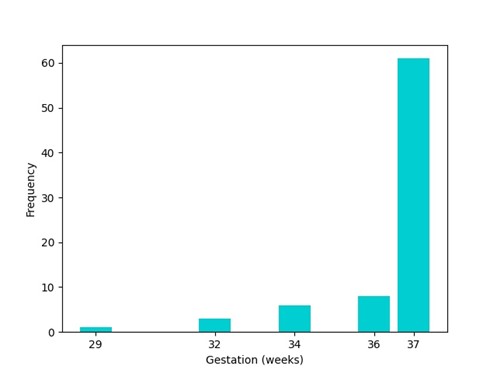


**Figure S1**. Distribution of gestation (in weeks) threshold applied for PTB in omics biomarker studies identified in this review. This plot was generated using ‘Matplotlib’ v3.3.3 package in Python 3.8.

**References**

1. Pandey M., Awasthi S. and Baranwal S. (2020) IL-6: An endogenous activator of MMP-9 in preterm birth. *J Reprod Immunol* **141**, 103147.
2. Hao Y., Yan L., Pang Y*.* et al. (2020) Maternal serum level of manganese, single nucleotide polymorphisms, and risk of spontaneous preterm birth: A nested case-control study in China. *Environmental Pollution* **262**, 114187.
3. Gillespie S.L., Neal J.L., Christian L.M. et al. (2017) Interleukin-1 Receptor Antagonist Polymorphism and Birth Timing: Pathway Analysis Among African American Women. *Nurs Res* **66**, 95-104.
4. Frey H.A., Stout M.J., Pearson L.N. et al. (2016) Genetic variation associated with preterm birth in African-American women. *Am J Obstet Gynecol* **215**, 235.e231–235.e2358.
5. Langmia I.M., Apalasamy Y.D., Omar S.Z. et al. (2016) Interleukin 1 receptor type 2 gene polymorphism is associated with reduced risk of preterm birth. *J Matern Fetal Neonatal Med* **29**, 3347-3350.
6. Ramos B.R., Mendes N.D., Tanikawa A.A. et al. (2016) Ancestry informative markers and selected single nucleotide polymorphisms in immunoregulatory genes on preterm labor and preterm premature rupture of membranes: a case control study. *BMC Pregnancy Childbirth* **16**, 30.
7. Bream E.N., Leppellere C.R., Cooper M.E. et al. (2013) Candidate gene linkage approach to identify DNA variants that predispose to preterm birth. *Pediatr Res* **73**, 135-141.
8. Falah N., McElroy J., Snegovskikh V. et al. (2013) Investigation of genetic risk factors for chronic adult diseases for association with preterm birth. *Hum Genet* **132**, 57-67.
9. Harmon Q.E., Engel S.M., Olshan A.F. et al. (2013) Association of polymorphisms in natural killer cell-related genes with preterm birth. *Am J Epidemiol* **178**, 1208-1218.
10. Iwanaga R., Sugita N., Hirano E. et al. (2011) FcgammaRIIB polymorphisms, periodontitis and preterm birth in Japanese pregnant women. *J Periodontal Res* **46**, 292-302.
11. Romero R., Velez Edwards D.R., Kusanovic J.P. et al. (2010) Identification of fetal and maternal single nucleotide polymorphisms in candidate genes that predispose to spontaneous preterm labor with intact membranes. *Am J Obstet Gynecol* **202**, 431.e431–431.e4334.
12. Mustafa M.D., Pathak R., Ahmed T. et al. (2010) Association of glutathione S-transferase M1 and T1 gene polymorphisms and oxidative stress markers in preterm labor. *Clin Biochem* **43**, 1124-1128.
13. Romero R., Friel L.A., Velez Edwards D.R. et al. (2010) A genetic association study of maternal and fetal candidate genes that predispose to preterm prelabor rupture of membranes (PROM). *Am J Obstet Gynecol* **203**, 361.e361–361.e330.
14. Kwon H.S., Sohn I.S., Lee J.Y. et al. (2009) Intercellular adhesion molecule-1 K469E polymorphism in Korean patients with spontaneous preterm delivery. *Int J Gynaecol Obstet* **104**, 37-39.
15. Gebhardt S., Bruiners N. and Hillermann R. (2009) A novel exonic variant (221delT) in the LGALS13 gene encoding placental protein 13 (PP13) is associated with preterm labour in a low risk population. *J Reprod Immunol* **82**, 166-173.
16. Menon R., Velez D., Morgan N. et al. (2008) Genetic regulation of amniotic fluid TNF-alpha and soluble TNF receptor concentrations affected by race and preterm birth. *Human genetics* **124**, 243-253.
17. Velez D.R., Fortunato S.J., Thorsen P. et al. (2008) Preterm birth in Caucasians is associated with coagulation and inflammation pathway gene variants. *PLoS One* **3**, e3283.
18. Steffen K.M., Cooper M.E., Shi M. et al. (2007) Maternal and fetal variation in genes of cholesterol metabolism is associated with preterm delivery. *J Perinatol* **27**, 672-680.
19. Ehn N.L., Cooper M.E., Orr K. et al. (2007) Evaluation of fetal and maternal genetic variation in the progesterone receptor gene for contributions to preterm birth. *Pediatr Res* **62**, 630-635.
20. Menon R., Velez D.R., Simhan H. et al. (2006) Multilocus interactions at maternal tumor necrosis factor-alpha, tumor necrosis factor receptors, interleukin-6 and interleukin-6 receptor genes predict spontaneous preterm labor in European-American women. *Am J Obstet Gynecol* **194**, 1616-1624.
21. Kalish R.B., Vardhana S., Normand N.J. et al. (2006) Association of a maternal CD14 -159 gene polymorphism with preterm premature rupture of membranes and spontaneous preterm birth in multi-fetal pregnancies. *J Reprod Immunol* **70**, 109-117.
22. Kalish R.B., Nguyen D.P., Vardhana S. et al. (2005) A single nucleotide A>G polymorphism at position -670 in the Fas gene promoter: relationship to preterm premature rupture of fetal membranes in multifetal pregnancies. *Am J Obstet Gynecol* **192**, 208-212.
23. Annells M.F., Hart P.H., Mullighan C.G. et al. (2004) Interleukins-1, -4, -6, -10, tumor necrosis factor, transforming growth factor-beta, FAS, and mannose-binding protein C gene polymorphisms in Australian women: Risk of preterm birth. *Am J Obstet Gynecol* **191**, 2056-2067.
24. Hartel C., Finas D., Ahrens P. et al. (2004) Polymorphisms of genes involved in innate immunity: association with preterm delivery. *Mol Hum Reprod* **10**, 911-915.
25. Valdez L.L., Quintero A., Garcia E. et al. (2004) Thrombophilic polymorphisms in preterm delivery. *Blood Cells Mol Dis* **33**, 51-56.
26. Miller R., Smiley R., Thom E.A. et al. (2015) The association of beta-2 adrenoceptor genotype with short-cervix mediated preterm birth: a case-control study. *BJOG* **122**, 1387-1394.
27. Schmid M., Grimm C., Leipold H. et al. (2010) A polymorphism of the corticotropin-releasing hormone receptor 2 (CRHR2) and preterm birth. *Dis Markers* **28**, 37-42.
28. Uvuz F., Kilic S., Yilmaz N. et al. (2009) Relationship between preterm labor and thrombophilic gene polymorphism: A prospective sequential cohort study. *Gynecol Obstet Invest* **68**, 234-238.
29. Wang L.K., Huang M.C., Liu C.C. et al. (2017) Second-trimester plasma mannose-binding lectin levels and risk of preterm birth. *J Matern Fetal Neonatal Med* **30**, 678-683.
30. Fortunato S.J., Menon R., Velez D.R. et al. (2008) Racial disparity in maternal-fetal genetic epistasis in spontaneous preterm birth. *Am J Obstet Gynecol* **198**.
31. Manuck T.A., Lai Y., Meis P.J. et al. (2011) Admixture mapping to identify spontaneous preterm birth susceptibility loci in African Americans. *Obstet Gynecol* **117**, 1078-1084.
32. Zhou, G., Holzman, C., Chen, B. et al. (2021) EBF1-Correlated Long Non-coding RNA Transcript Levels in 3rd Trimester Maternal Blood and Risk of Spontaneous Preterm Birth. *Reproductive sciences* **28**, 541–549.
33. Zhou G., Holzman C., Heng Y.J. et al. (2020) Maternal blood EBF1-based microRNA transcripts as biomarkers for detecting risk of spontaneous preterm birth: a nested case-control study. *J Matern Fetal Neonatal Med* 1-9.
34. Awasthi S. and Pandey M. (2019) Association of TLR4 and TNF-α Gene Polymorphisms and TLR4 mRNA Levels in Preterm Birth in a Northern Indian Population. *Indian Pediatrics* **56**, 202–204.
35. Mustafa M., Garg N., Banerjee B.D. et al. (2015) Inflammatory-mediated pathway in association with organochlorine pesticides levels in the etiology of idiopathic preterm birth. *Reprod Toxicol* **57**, 111-120.
36. Lee S.Y., Buhimschi I.A., Dulay A.T. et al. (2011) IL-6 trans-signaling system in intra-amniotic inflammation, preterm birth, and preterm premature rupture of the membranes. *J Immunol* **186**, 3226-3236.
37. Afzal A., Ashraf S. and Ashraf M. (2017) Maternal Plasma C-Reactive Protein: A Predictive Tool for Preterm Delivery. *Journal of Evolution of Medical and Dental Sciences* **6**, 369-374.
38. Bakalis S.P., Poon L.C., Vayna A.M. et al. (2012) C-reactive protein at 11-13 weeks' gestation in spontaneous early preterm delivery. *J Matern Fetal Neonatal Med* **25**, 2475-2478.
39. Berkowitz G., Lapinski R., Lockwood C. et al. (1996) Corticotropin-releasing factor and its binding protein: Maternal serum levels in term and preterm deliveries. *American journal of obstetrics and gynecology* **174**, 1477–1483.
40. Bernstein P., Stern R., Lin N. et al. (1998) Beta-human chorionic gonadotropin in cervicovaginal secretions as a predictor of preterm delivery. *Am J Obstet Gynecol* **179**.
41. Beta J., Bredaki F.E., Rodriguez Calvo J. et al. (2011) Maternal serum alpha-fetoprotein at 11-13 weeks' gestation in spontaneous early preterm delivery. *Fetal Diagn Ther* **30**, 88-93.
42. Biggio J.R., Jr., Ramsey P.S., Cliver S.P. et al. (2005) Midtrimester amniotic fluid matrix metalloproteinase-8 (MMP-8) levels above the 90th percentile are a marker for subsequent preterm premature rupture of membranes. *Am J Obstet Gynecol* **192**, 109-113.
43. Cantonwine D.E., Zhang Z., Rosenblatt K. et al. (2016) Evaluation of proteomic biomarkers associated with circulating microparticles as an effective means to stratify the risk of spontaneous preterm birth. *Am J Obstet Gynecol* **214**, 631.e631–631.e611.
44. Catov J.M., Bertolet M., Chen Y.F. et al. (2014) Nonesterified fatty acids and spontaneous preterm birth: a factor analysis for identification of risk patterns. *Am J Epidemiol* **179**, 1208-1215.
45. Curry A.E., Thorsen P., Drews C. et al. (2009) First-trimester maternal plasma cytokine levels, pre-pregnancy body mass index, and spontaneous preterm delivery. *Acta Obstet Gynecol Scand* **88**, 332-342.
46. Curry A.E., Vogel I., Drews C. et al. (2007) Mid-pregnancy maternal plasma levels of interleukin 2, 6, and 12, tumor necrosis factor-alpha, interferon-gamma, and granulocyte-macrophage colony-stimulating factor and spontaneous preterm delivery. *Acta Obstet Gynecol Scand* **86**, 1103-1110.
47. Dane B., Dane C., Batmaz G. et al. (2013) First trimester maternal serum pregnancy-associated plasma protein-A is a predictive factor for early preterm delivery in normotensive pregnancies. *Gynecol Endocrinol* **29**, 592-595.
48. Dunn A.B., Dunlop A.L., Miller A.H. et al. (2019) Complement Activation During Early Pregnancy and Clinical Predictors of Preterm Birth in African American Women. *J Perinat Neonatal Nurs* **33**, E15-E26.
49. Ellis M., Livesey J., Inder W. et al. (2002) Plasma corticotropin-releasing hormone and unconjugated estriol in human pregnancy: Gestational patterns and ability to predict preterm delivery. *American journal of obstetrics and gynecology* **186**, 94–99.
50. Esplin M.S., Merrell K., Goldenberg R. et al. (2011) Proteomic identification of serum peptides predicting subsequent spontaneous preterm birth. *Am J Obstet Gynecol* **204**, 391.e391–391.e3918.
51. Ferguson K.K., McElrath T.F., Chen Y.H. et al. (2014) Longitudinal profiling of inflammatory cytokines and C-reactive protein during uncomplicated and preterm pregnancy. *Am J Reprod Immunol* **72**, 326-336.
52. Forest J., Masse J. and Moutquin J. (1996) Maternal hematocrit and albumin as predictors of intrauterine growth retardation and preterm delivery. *Clinical Biochemistry* **29**, 563–566.
53. Hallingstrom M., Cobo T., Kacerovsky M. et al. (2020) The association between selected mid-trimester amniotic fluid candidate proteins and spontaneous preterm delivery. *J Matern Fetal Neonatal Med* **33**, 583-592.
54. Hallingstrom M., Lenco J., Vajrychova M. et al. (2016) Proteomic Analysis of Early Mid-Trimester Amniotic Fluid Does Not Predict Spontaneous Preterm Delivery. *PLoS One* **11**, e0155164.
55. Holzman C., Jetton J., Siler-Khodr T. et al. (2001) Second trimester corticotropin-releasing hormone levels in relation to preterm delivery and ethnicity. *Obstetrics and gynecology* **97**, 657-663.
56. Holzman C., Senagore P.K. and Wang J. (2013) Mononuclear leukocyte infiltrate in extraplacental membranes and preterm delivery. *Am J Epidemiol* **177**, 1053-1064.
57. Hudic I., Szekeres-Bartho J., Stray-Pedersen B. et al. (2016) Lower Urinary and Serum Progesterone-Induced Blocking Factor in Women with Preterm Birth. *J Reprod Immunol* **117**, 66-69.
58. Hunter P.J., Sheikh S., David A.L. et al. (2016) Cervical leukocytes and spontaneous preterm birth. *J Reprod Immunol* **113**, 42-49.
59. Kaijomaa M., Ulander V.M., Hamalainen E. et al. (2016) The risk of adverse pregnancy outcome among pregnancies with extremely low maternal PAPP-A. *Prenat Diagn* **36**, 1115-1120.
60. Kalinka J., Sobala W., Wasiela M. et al. (2005) Decreased proinflammatory cytokines in cervicovaginal fluid, as measured in midgestation, are associated with preterm delivery. *Am J Reprod Immunol* **54**, 70-76.
61. Kallioniemi H., Rahkonen L., Heikinheimo O. et al. (2013) Early pregnancy vaginal fluid phosphorylated insulin-like growth factor binding protein-1 predicts preterm delivery. *Prenat Diagn* **33**, 378-383.
62. Kansu-Celik H., Tasci Y., Karakaya B.K. et al. (2019) Maternal serum advanced glycation end products level as an early marker for predicting preterm labor/PPROM: a prospective preliminary study. *J Matern Fetal Neonatal Med* **32**, 2758-2762.
63. Kesrouani A., Chalhoub E., El Rassy E. et al. (2016) Prediction of preterm delivery by second trimester inflammatory biomarkers in the amniotic fluid. *Cytokine* **85**. 67-70.
64. Kim A., Lee E.S., Shin J.C. et al. (2013) Identification of biomarkers for preterm delivery in mid-trimester amniotic fluid. *Placenta* **34**, 873-878.
65. Kim Y.H., Park Y.W., Kwon H.S. et al. (2005) Vaginal fluid beta-human chorionic gonadotropin level in the diagnosis of premature rupture of membranes. *Acta Obstet Gynecol Scand.* **84**, 802-5.
66. Kirkegaard I., Henriksen T.B., Torring N. et al. (2011) PAPP-A and free beta-hCG measured prior to 10 weeks is associated with preterm delivery and small-for-gestational-age infants. *Prenat Diagn* **31**, 171-175.
67. Kirkegaard I., Uldbjerg N., Petersen O.B. et al. (2010) PAPP-A, free beta-hCG, and early fetal growth identify two pathways leading to preterm delivery. *Prenat Diagn* **30**, 956-963.
68. Kramer M.S., Kahn S.R., Platt R.W. et al. (2010) Mid-trimester maternal plasma cytokines and CRP as predictors of spontaneous preterm birth. *Cytokine* **49**, 10-14.
69. Kumar S., Palaia T., Hall C.E. et al. (2015) Role of Lipocalin-type prostaglandin D2 synthase (L-PGDS) and its metabolite, prostaglandin D2, in preterm birth. *Prostaglandins Other Lipid Mediat* **118-119**, 28-33.
70. Lee S.E., Kim S.C., Kim K.H. et al. (2016) Detection of angiogenic factors in midtrimester amniotic fluid and the prediction of preterm birth. *Taiwan J Obstet Gynecol* **55**, 539-544.
71. Malamitsi-Puchner A., Vrachnis N., Samoli E. et al. (2006) Investigation of midtrimester amniotic fluid factors as potential predictors of term and preterm deliveries. *Mediators Inflamm* **2006**: 94381.
72. Manning R., James C.P., Smith M.C. et al. (2019) Predictive value of cervical cytokine, antimicrobial and microflora levels for pre-term birth in high-risk women. *Sci Rep* **9**, 11246.
73. Massaro G., Scaravilli G., Simeone S. et al. (2009) Interleukin-6 and Mycoplasma hominis as markers of preterm birth and related brain damage: our experience. *J Matern Fetal Neonatal Med* **22**, 1063-1067.
74. McDonald C.R., Darling A.M., Conroy A.L. et al. (2015) Inflammatory and Angiogenic Factors at Mid-Pregnancy Are Associated with Spontaneous Preterm Birth in a Cohort of Tanzanian Women. *PLoS One* **10**, e0134619.
75. McElrath T.F., Cantonwine D.E., Jeyabalan A. et al. (2019) Circulating microparticle proteins obtained in the late first trimester predict spontaneous preterm birth at less than 35 weeks' gestation: a panel validation with specific characterization by parity. *Am J Obstet Gynecol* **220**, 488.e481–488.e411.
76. Mijal R.S., Holzman C.B., Rana S. et al. (2012) Mid-pregnancy levels of angiogenic markers as indicators of pathways to preterm delivery. *J Matern Fetal Neonatal Med* **25**, 1135-1141.
77. Moawad A.H., Goldenberg R.L., Mercer B. et al. (2002) The Preterm Prediction Study: The value of serum alkaline phosphatase, α-fetoprotein, plasma corticotropin-releasing hormone, and other serum markers for the prediction of spontaneous preterm birth. *American Journal of Obstetrics and Gynecology* **186**, 990-996.
78. Neggers Y., Goldenberg R., DuBard M. et al. (2000) Increased risk of preterm delivery with elevated maternal alpha-fetoprotein and plasma zinc levels in African-American women. *Acta Obstet Gynecol Scand* **79**, 160–164.
79. Ong C., Liao A., Spencer K. et al. (2000) First trimester maternal serum free beta human chorionic gonadotrophin and pregnancy associated plasma protein A as predictors of pregnancy complications. *British Journal of Obstetrics and Gynaecology* **107**, 1265–1270.
80. Ozer K.T., Kavak Z.N., Gokaslan H. et al. (2005) Predictive power of maternal serum and amniotic fluid CRP and PAPP-A concentrations at the time of genetic amniocentesis for the preterm delivery. *Eur J Obstet Gynecol Reprod Biol* **122**, 187-190.
81. Ozgu-Erdinc A.S., Cavkaytar S., Aktulay A. et al. (2014) Mid-trimester maternal serum and amniotic fluid biomarkers for the prediction of preterm delivery and intrauterine growth retardation. *J Obstet Gynaecol Res* **40**, 1540-1546.
82. Parry S., Leite R., Esplin M.S. et al. (2020) Cervicovaginal fluid proteomic analysis to identify potential biomarkers for preterm birth. *Am J Obstet Gynecol* **222**, 493.e491–493.e413.
83. Parry S., Zhang H., Biggio J. et al. (2014) Maternal serum serpin B7 is associated with early spontaneous preterm birth. *Am J Obstet Gynecol* **211**, 678.e671–678.e612.
84. Pawelczyk E., Nowicki B., Izban M. et al. (2010) Spontaneous preterm labor is associated with an increase in the proinflammatory signal transducer TLR4 receptor on maternal blood monocytes. *BMC pregnancy and childbirth* **10**: 66.
85. Pearce B.D., Grove J., Bonney E.A. et al. (2010) Interrelationship of cytokines, hypothalamic-pituitary-adrenal axis hormones, and psychosocial variables in the prediction of preterm birth. *Gynecol Obstet Invest* **70**, 40-46.
86. Pihl K., Larsen T., Laursen I. et al. (2009) First trimester maternal serum pregnancy-specific beta-1-glycoprotein (SP1) as a marker of adverse pregnancy outcome. *Prenat Diagn* **29**, 1256-1261.
87. Pitiphat W., Gillman M.W., Joshipura K.J. et al. (2005) Plasma C-reactive protein in early pregnancy and preterm delivery. *Am J Epidemiol* **162**, 1108-1113.
88. Puchner K., Iavazzo C., Gourgiotis D. et al. (2011) Mid-trimester amniotic fluid interleukins (IL-1β, IL-10 and IL-18) as possible predictors of preterm delivery. *In Vivo* **25**, 141–148.
89. Puchner K., Iavazzo C., Gourgiotis D. et al. (2012) The implication of second-trimester amniotic fluid TNF-alpha, cytochrome C and cell death nucleosomes in the prediction of preterm labor and/or premature rupture of membranes. *Arch Gynecol Obstet* **285**, 37-43.
90. Rahkonen L., Rutanen E.M., Nuutila M. et al. (2010) Elevated levels of decidual insulin-like growth factor binding protein-1 in cervical fluid in early and mid-pregnancy are associated with an increased risk of spontaneous preterm delivery. *BJOG* **117**, 701-710.
91. Rosen T., Kuczynski E., O'Neill L.M. et al. (2001) Plasma levels of thrombin-antithrombin complexes predict preterm premature rupture of the fetal membranes. *Journal of Maternal-Fetal and Neonatal Medicine* **10**, 297-300.
92. Ruiz R.J., Gennaro S., O'Connor C. et al. (2016) CRH as a Predictor of Preterm Birth in Minority Women. *Biol Res Nurs* **18**, 316-321.
93. Ruiz R.J., Jallo N., Murphey C. et al. (2012) Second trimester maternal plasma levels of cytokines IL-1Ra, Il-6 and IL-10 and preterm birth. *J Perinatol* **32**, 483-490.
94. Saade G.R., Boggess K.A., Sullivan S.A. et al. (2016) Development and validation of a spontaneous preterm delivery predictor in asymptomatic women. *Am J Obstet Gynecol* **214**, 633 e631-633 e624.
95. Shin J.E., Shin J.C., Kim S.J. et al. (2016) Early midtrimester serum insulin-like factors and cervical length to predict preterm delivery. *Taiwan J Obstet Gynecol* **55**, 45-49.
96. Smith G., Stenhouse E., Crossley J. et al. (2002) Early Pregnancy Levels of Pregnancy-Associated Plasma Protein A and the Risk of Intrauterine Growth Restriction, Premature Birth, Preeclampsia, and Stillbirth. *The Journal of Clinical Endocrinology & Metabolism* **87**, 1762–1767.
97. Soni S., Krantz D.A., Blitz M.J. et al. (2019) Elevated maternal serum-free beta-human chorionic gonadotropin (beta-hCG) and reduced risk of spontaneous preterm delivery. *J Matern Fetal Neonatal Med* **32**, 3191-3196.
98. Sorokin Y., Romero R., Mele L. et al. (2010) Maternal serum interleukin-6, C-reactive protein, and matrix metalloproteinase-9 concentrations as risk factors for preterm birth <32 weeks and adverse neonatal outcomes. *Am J Perinatol* **27**, 631-640.
99. Straughen J.K., Kumar P. and Misra V.K. (2012) The effect of maternal soluble FMS-like tyrosine kinase 1 during pregnancy on risk of preterm delivery. *J Matern Fetal Neonatal Med* **25**, 1879-1883.
100. Tamura T., Goldenberg R., Johnston K. et al. (1996) Serum ferritin: A predictor of early spontaneous preterm delivery. *Obstetrics and gynecology* **87**, 360-365.
101. Thorsen P., Schendel D., Deshpande A. et al. (2001) Identification of biological biochemical marker(s) for preterm delivery. *Paediatric and perinatal epidemiology* **15**, 90–103.
102. Vogel I., Thorsen P., Hundborg H.H. et al. (2006) Prediction of preterm delivery using changes in serum relaxin in low risk pregnancies. *Eur J Obstet Gynecol Reprod Biol* **128**, 113-118.
103. Wallenstein M.B., Jelliffe-Pawlowski L.L., Yang W. et al. (2016) Inflammatory biomarkers and spontaneous preterm birth among obese women. *J Matern Fetal Neonatal Med* **29**, 3317-3322.
104. Whitcomb B.W., Schisterman E.F., Luo X. et al. (2009) Maternal serum granulocyte colony-stimulating factor levels and spontaneous preterm birth. *J Womens Health (Larchmt)* **18**, 73-78.
105. Zhang J., Shynlova O., Sabra S. et al. (2017) Immunophenotyping and activation status of maternal peripheral blood leukocytes during pregnancy and labour, both term and preterm. *J Cell Mol Med* **21**, 2386-2402.
106. Zhu H. and Yang M.J. (2018) Maternal plasma concentrations of macrophage migration inhibitory factor at first trimester as a predictive biomarker of preterm delivery in Chinese women. *Clin Chim Acta* **483**, 286-290.
107. Patil A.S., Gaikwad N.W., Grotegut C.A. et al. (2020) Alterations in endogenous progesterone metabolism associated with spontaneous very preterm delivery. *Hum Reprod Open* **2020**, hoaa007.
108. Eick S.M., Ferguson K.K., Milne G.L. et al. (2020) Repeated measures of urinary oxidative stress biomarkers and preterm birth in Puerto Rico. *Free Radic Biol Med* **146**, 299-305.
109. Rosen E.M., van 't Erve T.J., Boss J. et al. (2019) Urinary oxidative stress biomarkers and accelerated time to spontaneous delivery. *Free Radic Biol Med* **130**, 419-425.
110. Ferguson K.K., McElrath T.F., Ko Y.A. et al. (2014) Variability in urinary phthalate metabolite levels across pregnancy and sensitive windows of exposure for the risk of preterm birth. *Environ Int* **70**, 118-124.
111. Giannella L., Giulini S., Cerami L.B. et al. (2011) Periodontal disease and nitric oxide levels in low risk women with preterm labor. *Eur J Obstet Gynecol Reprod Biol* **158**, 47-51.
112. Adibi J.J., Hauser R., Williams P.L. et al. (2009) Maternal urinary metabolites of Di-(2-Ethylhexyl) phthalate in relation to the timing of labor in a US multicenter pregnancy cohort study. *Am J Epidemiol* **169**, 1015-1024.
113. Longini M., Perrone S., Vezzosi P. et al. (2007) Association between oxidative stress in pregnancy and preterm premature rupture of membranes. *Clin Biochem* **40**, 793-797.
114. Fabregat A., Jupe S., Matthews L. et al. (2018) The Reactome Pathway Knowledgebase. *Nucleic Acids Res* **46**, D649-D655.
115. Fabregat A., Sidiropoulos K., Viteri G. et al. (2017) Reactome pathway analysis: a high-performance in-memory approach. *BMC Bioinformatics* **18**, 142.
